# METTL3 is essential for postnatal development of brown adipose tissue and energy expenditure in mice

**DOI:** 10.1038/s41467-020-15488-2

**Published:** 2020-04-03

**Authors:** Yuqin Wang, Ming Gao, Fuxing Zhu, Xinzhi Li, Ying Yang, Qiuxin Yan, Linna Jia, Liwei Xie, Zheng Chen

**Affiliations:** 1 HIT Center for Life Sciences, School of Life Science and Technology, Harbin Institute of Technology, Harbin, 150001 China; 2 Key Laboratory of Molecular Epigenetics of the Ministry of Education (MOE), School of Life Sciences, Northeast Normal University, Changchun, 130024 China; 3 State Key Laboratory of Applied Microbiology Southern China, Guangdong Provincial Key Laboratory of Microbial Culture Collection and Application, Guangdong Open Laboratory of Applied Microbiology, Guangdong Institute of Microbiology, Guangdong Academy of Sciences, Guangzhou, 510070 China

**Keywords:** Endocrine system and metabolic diseases, Endocrine system and metabolic diseases

## Abstract

Brown adipose tissue (BAT) undergoes rapid postnatal development and then protects against cold and obesity into adulthood. However, the molecular mechanism that determines postnatal development and maturation of BAT is largely unknown. Here we show that METTL3 (a key RNA methyltransferase) expression increases significantly in interscapular brown adipose tissue (iBAT) after birth and plays an essential role in the postnatal development and maturation of iBAT. BAT-specific deletion of *Mettl3* severely impairs maturation of BAT in vivo by decreasing m^6^A modification and expression of *Prdm16*, *Pparg*, and *Ucp1* transcripts, which leads to a marked reduction in BAT-mediated adaptive thermogenesis and promotes high-fat diet (HFD)-induced obesity and systemic insulin resistance. These data demonstrate that METTL3 is an essential regulator that controls iBAT postnatal development and energy homeostasis.

## Abstract (web summary)

N6-methyladenosine (m^6^A) is one of the most prevalent mRNA modifications in eukaryotes. Here the authors show that the m^6^A writer protein methyltransferase-like (METTL) 3 plays an important role for the development of brown adipose tissue.

## Introduction

Obesity and its associated diseases affect billions of people around the world. Obesity results from an imbalance between energy intake and energy expenditure. Increasing energy expenditure is an efficient way to treat obesity^1–3^. Brown adipose tissue (BAT) dissipates the mitochondrial electrochemical gradient to generate heat through uncoupling protein1 (UCP1)^1,4^. It has been shown that adult humans also have functional UCP1-positive brown adipocytes^5–7^, and activation of BAT by cold stimuli leads to a decrease in fat mass^8^. Brown adipocytes undergo postnatal development to mature and gain function^9,10^. Thus, identification of targetable factors that promote postnatal development and function of BAT is an attractive strategy for treating obesity.

It has been demonstrated that PRDM16 and PPARγ are early key transcriptional factors in the fate-determination of brown fat^10–13^. In addition, PGC-1α expression is induced by cold challenge or by β3-adrenergic agonists^14,15^. PGC-1α then triggers mitochondrial biogenesis, oxidative metabolism, and thermogenesis by inducing expression of *Ucp1* and many other genes^14,15^. Whether their mRNA N6-methyladenosine (m^6^A) modification regulates BAT development and energy expenditure is largely unknown.

m^6^A is one of the most prevalent mRNA modifications in eukaryotes^16^. RNA m^6^A modification can be catalyzed by m^6^A writer proteins (METTL3/METTL14/WTAP)^17,18^, recognized by m^6^A reader proteins (YTHDF1-3)^19,20^, and removed by m^6^A eraser proteins (FTO and ALKBH5)^21,22^. Recent studies have demonstrated that m^6^A modification regulates most RNA processing steps, including mRNA splicing^23^, mRNA stability^24^, translation efficiency^25^, microRNA maturation^26^, and X chromosome imprinting^27^ by m^6^A writer, reader, and eraser proteins, which further modulate a variety of biological processes such as circadian rhythms^28^, DNA damage response^29^, stem cell differentiation^30,31^, and white fat cell differentiation^32,33^. Methyltransferase-like 3 (METTL3), a key RNA methyltransferase, has been demonstrated to regulate neurogenesis^34^, spermatogenesis^35,36^, early embryonic development^31^, stem cell pluripotency in mice^30,31^, and white fat cell differentiation in vitro^37^. However, whether METTL3-mediated m^6^A modification of mRNA regulates interscapular brown adipose tissue (iBAT) postnatal development and thermogenesis is not investigated.

Here, we report that METTL3 is an essential iBAT-enriched RNA methyltransferase and controls iBAT postnatal development and maturation by regulating m^6^A modification and expression of *Prdm16*, *Pparg*, and *Ucp1* transcripts. BAT-specific knockout of *Mettl3* leads to a marked reduction in BAT-mediated adaptive thermogenesis and further promotes high-fat diet (HFD)-induced obesity and systemic insulin resistance. These data demonstrate that METTL3 is an essential regulator that controls iBAT postnatal development and energy homeostasis.

## Results

### METTL3 is enriched in iBAT and associated with postnatal development of iBAT

**Fig. 1:**
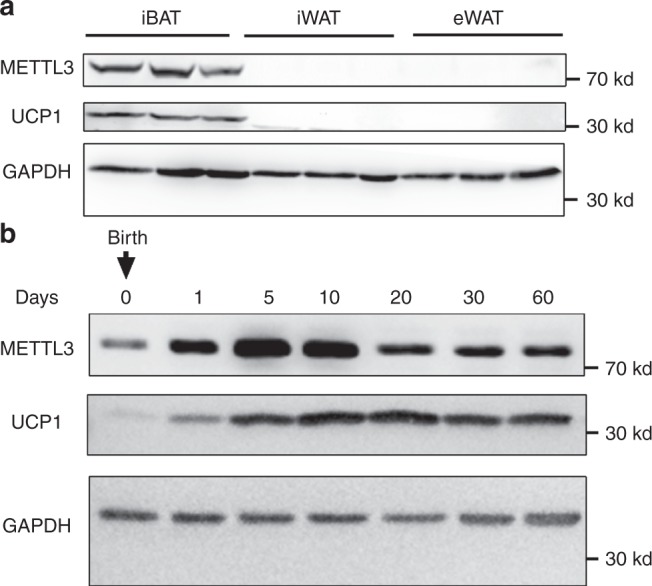
METTL3 is enriched in iBAT and associated with postnatal development of iBAT. **a** METTL3 and UCP1 protein levels in iBAT, iWAT, and eWAT of C57BL/6 male mice at 8 weeks of age. **b** METTL3 and UCP1 protein levels in iBAT at 0, 1, 5, 10, 20, 30, and 60 days after birth. These experiments were repeated three times independently with similar results. Source data are provided as a Source Data file.

First, we observed that METTL3 was highly enriched in interscapular BAT (iBAT) compared with that in inguinal white adipose tissue (iWAT) and epididymal white adipose tissue (eWAT) (Fig. 1a), indicating that METTL3 may regulate the function of iBAT. Next, to determine whether METTL3 is associated with the postnatal development of iBAT, we measured METTL3 expression in iBAT at different ages after birth. METTL3 protein levels were dramatically increased in iBAT at 1 day of age, reached the maximal level at 5 days of age, and began a slight decline after 10 days of age (Fig. 1b). As expected, the UCP1 levels were highly induced in iBAT at 1 day of age, reached the maximal level between 10 and 20 days of age, and began a slight decline after 20 days of age (Fig. 1b). These data demonstrate that METTL3 expression shows a similar expression pattern with UCP1 in iBAT during early development after birth, indicating that METTL3 may have an important role in the postnatal development of iBAT.

### METTL3 is essential for postnatal development of iBAT

**Fig. 2:**
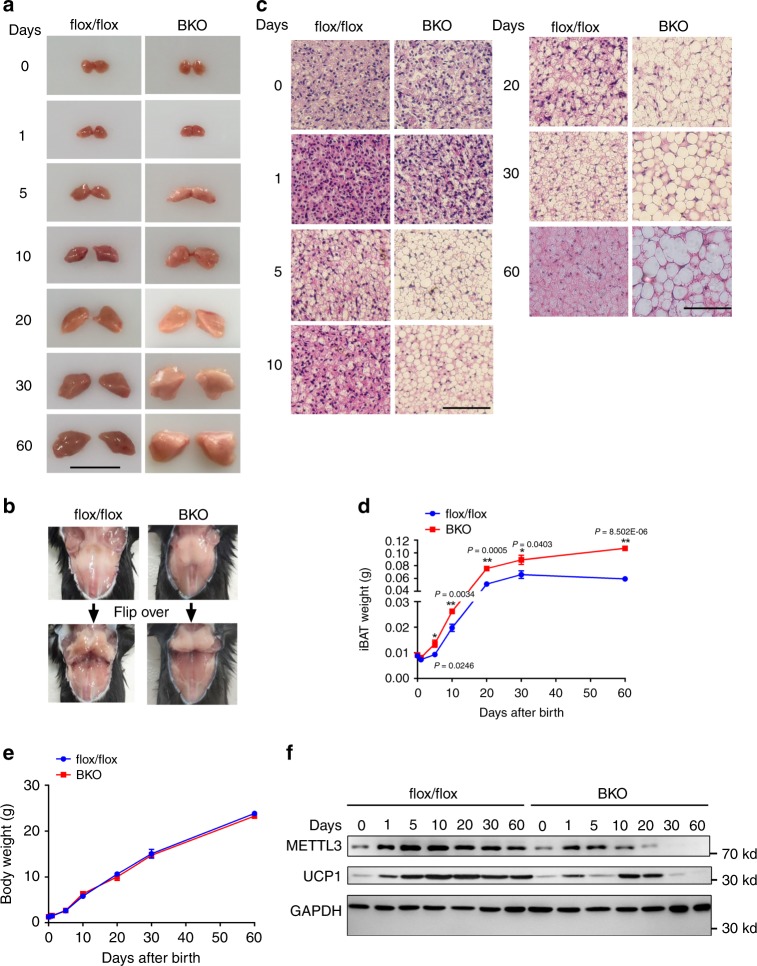
METTL3 is required for postnatal development of iBAT. **a** Gross appearance of iBATs in *Mettl3*^flox/flox^ and BKO mice at 0, 1, 5, 10, 20, 30, and 60 days after birth. The scale bar represents 1 cm. **b** Gross appearance of iBATs in *Mettl3*^flox/flox^ and BKO mice at 8 weeks of age. **c** Hematoxylin and eosin (H&E) staining of iBATs in *Mettl3*^flox/flox^ and BKO mice at 0, 1, 5, 10, 20, 30, and 60 days after birth. Scale bars represent 100 μm. Three mice for each group were used for H&E staining with similar results. **d**, **e** iBAT and body weight of METTL3 ^flox/flox^ and BKO mice at 0, 1, 5, 10, 20, 30, and 60 days after birth (*n* = 5–8 for each group). **f** METTL3 and UCP1 protein levels in iBAT of *Mettl3*^flox/flox^ and BKO mice at 0, 1, 5, 10, 20, 30, and 60 days after birth. This immunoblotting experiment was repeated three times independently with similar results. Data represent the mean ± SEM. Significance was determined by unpaired two-tailed Student’s *t* test analysis. *p < 0.05. **p < 0.01. Source data are provided as a Source Data file.

To further determine whether METTL3 regulates iBAT postnatal development, we generated BAT-specific *Mettl3* knockout (BKO) mice by crossing *Mettl3*-floxed mice (Supplementary Fig. 1a–c) with *Ucp1*-iCre transgenic mice, in which IRES-Cre was inserted between exon 6 and the 3′ untranslated region (UTR) to allow *Ucp1* and iCre expression at the same time with lower levels^38^. *Ucp1* expression is induced after 18 days of gestation and shows a rapid increase until 10 days after birth^9^. Thus, *Ucp1*-iCre is supposed to delete genes in iBAT shortly after birth. METTL3 protein levels in iBAT of BKO mice began to decline at 5 days of age (see below). METTL3 was specifically deleted in iBAT but not in other tissues such as eWAT, liver, and skeletal muscle in 8-week-old BKO mice (Supplementary Fig. 1d). We also noted that METTL3 was highly enriched in iBAT compared with that in eWAT (Supplementary Fig. 1d). We did not observe any difference in body weight (Supplementary Fig. 2a), iBAT morphology (Supplementary Fig. 2b), iBAT weight (Supplementary Fig. 2c), or cold challenge (Supplementary Fig. 2d) between *Mettl3*^flox/flox^ and *Ucp1*-iCre mice. Therefore, we used *Mettl3*^flox/flox^ mice as the control for BKO mice in the following experiments. Surprisingly, the morphology of iBAT in BKO mice appeared abnormal, enlarged and “whitening” roughly after 5 days of age (Fig. 2a–c). Consistently, the weight of iBAT in BKO mice was significantly higher than that of flox/flox mice (Fig. 2d), which did not affect the body weight gain of BKO mice during postnatal development (Fig. 2e). The enlarged iBAT was attributable to large cytosolic lipid droplet accumulation, resulting in an increase in average adipocyte size (steatotic hypertrophy) (Fig. 2c). The key thermogenic protein, UCP1, was significantly increased during postnatal development in iBAT of flox/flox mice, whereas its induction was blocked in iBAT of BKO mice (Fig. 2f). The blockage of UCP1 induction in iBAT of BKO mice during postnatal development was associated with the decline of METTL3 in BKO mice after 5 days of age (Fig. 2f). These data indicate that METTL3 is necessary for postnatal development of iBAT.

### BAT-specific deletion of *Mettl3* results in dramatically decreased expression of BAT-selective genes

**Fig. 3:**
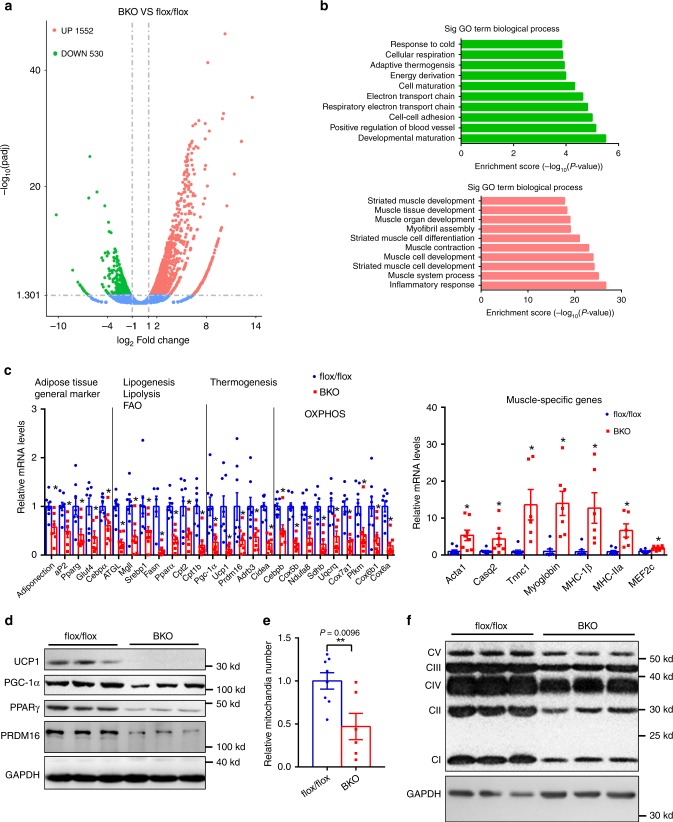
BAT-specific deletion of *Mettl3* dramatically decreases expression of BAT-selective genes RNA-seq analysis was performed in the iBATs of *Mettl3*^flox/flox^ and BKO mice at 8 weeks old. **a** The differentially expressed genes (DEGs) (BKO VS flox/flox) including 530 downregulated genes and 1552 upregulated genes were illustrated in a volcanoplot (fold change > 2 and *q* value < 0.005). **b** Top GO biological process terms enriched in downregulated and upregulated genes. **c** Relative mRNA levels (*n* = 5–8 for each group). **d** UCP1, PGC-1α, PPARγ, PRDM16, and GAPDH protein levels in iBATs of 8-week-old *Mettl3*^flox/flox^ and BKO mice were determined by Western blot (*n* = 3 for each group). **e** Relative mitochondria number in iBATs of 8-week-old *Mettl3*^flox/flox^ and BKO mice (*Mettl3*^flox/flox^, *n* = 8; BKO, *n* = 6). **f** Mitochondrial complex protein levels in iBATs of 8-week-old *Mettl3*^flox/flox^ and BKO mice were determined by western blot (*n* = 3 for each group). Data represent the mean ± SEM. Significance was determined by unpaired two-tailed Student’s *t* test analysis. *p < 0.05. **p < 0.01. Source data are provided as a Source Data file.

To further explore the molecular mechanisms of the impaired postnatal development of iBAT in BKO mice, we examined the whole transcriptional profiles of iBAT in both BKO and flox/flox mice by performing RNA sequencing (RNA-seq) analysis. As shown in Fig. 3a, 530 genes were downregulated, and 1552 genes were upregulated. Gene Ontology analysis showed that genes related to developmental maturation, respiratory electron transport chain, adaptive thermogenesis, and energy deprivation were dramatically downregulated, whereas genes associated with inflammation, muscle system process, and muscle cell development were significantly upregulated (Fig. 3b). Quantitative PCR analysis further supported these RNA-seq data. The general markers of iBAT, such as *adiponectin, aP2, Pparg, Glut4*, and *Cebpα*, were dramatically decreased in BKO mice (Fig. 3c). Lipogenesis-, lipolysis-, and fatty acid oxidation-related genes, including *Srebp1, Fasn, ATGL, MgII, Pparα, Cpt2*, and *Cpt1b*, were also decreased in BKO mice (Fig. 3c). Thermogenic genes, including *Pgc-1α, Ucp1, Prdm16, Adrb3*, and *Cidea*, were significantly downregulated (Fig. 3c). UCP1, PGC-1α, PPARγ, and PRDM16 protein levels were also dramatically reduced in iBAT of BKO mice (Fig. 3d). Decreased PGC-1α levels also resulted in reduced mitochondrial number (Fig. 3e). In addition, most of genes related to mitochondrial oxidative phosphorylation (OXPHOS), including the components of complexes I, II, III, IV, and V, were dramatically reduced in BKO mice (Fig. 3c, f). Interestingly, the genes associated with muscle system process and muscle cell development, including *Acta1*, *Casq2*, *Tnnc1*, *Myoglobin*, *MHC-1β*, *MHC-IIα,* and *MEF2c*, were dramatically increased in iBAT of BKO mice (Fig. 3c), indicating that BAT-specific deletion of *Mettl3* may promote the brown fat-myoblast conversion. These data also suggest that BAT-specific deletion of *Mettl3* impairs postnatal development of iBAT by decreasing expression of BAT-selective genes.

Lipid uptake has an important role in lipid accumulation and BAT function^39,40^. CD36 is the main fatty acid transporter in iBAT^39^. The lipoprotein lipase (LPL) and its inhibitor angiopoietin-like protein 4 (ANGPTL4) in iBAT controls cold-induced triglyceride uptake in BAT^39,40^. We measured the expression of *Cd36*, *Lpl*, and *Angptl4*. As shown in Supplementary Fig. 3, their mRNA levels were not altered in the iBAT of BKO mice. These data indicate that lipid uptake is not changed in the iBAT of BKO mice, which less likely contributes to the whitening of iBAT in BKO mice.

### METTL3 is necessary for m^6^A modification and expression of *Prdm16*, *Pparg*, and *Ucp1* transcripts

**Fig. 4:**
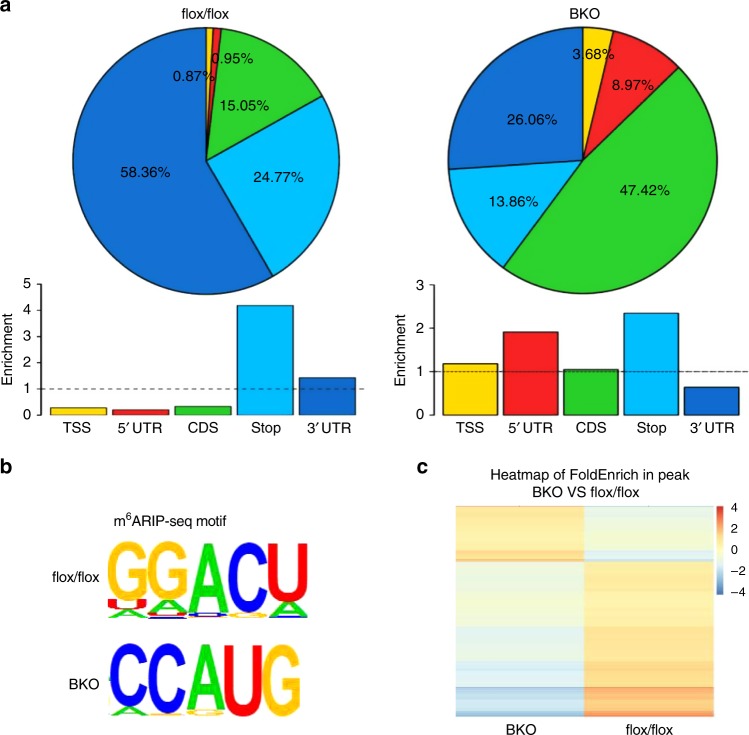
METTL3 is essential for mRNA m^6^A modification in iBAT. The m^6^A RNA immunoprecipitation sequencing (m^6^ARIP-seq) analysis of iBATs were performed in 8-week-old *Mettl3*^flox/flox^ and BKO mice. **a** The enrichment of m^6^ARIP-seq peaks in iBAT of 8-week-old *Mettl3*^flox/flox^ and BKO mice. **b** Consensus motif of m^6^A sites in iBAT of 8-week-old *Mettl3*^flox/flox^ and BKO mice. **c** Heatmap of FoldEnrich in m^6^A peaks (BKO VS flox/flox).

**Fig. 5:**
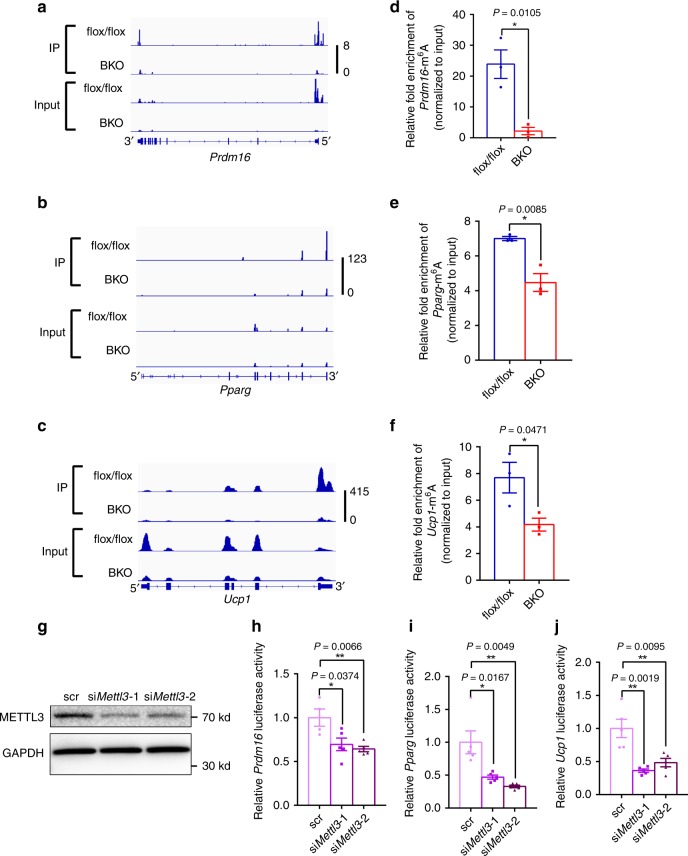
METTL3 is essential for m^6^A modification of *Prdm16*, *Pparg,* and *Ucp1* transcripts. **a**–**c** The read density from m^6^A-RIP-seq experiments on BKO–flox/flox pairs showing the m^6^A peaks identified in the *Prdm16*, *Pparg,* and *Ucp1* transcripts. **d**–**f** The m^6^A modification in *Prdm16*, *Pparg,* and *Ucp1* transcripts in iBAT of 8-week-old *Mettl3*^flox/flox^ and BKO mice were measured by m^6^ARIP-RT-qPCR (*n* = 3 for each group). **g**–**j** Primary brown preadipocytes seeded in 24-well plates were co-transfected with pMIR-REPORT Luciferase vectors (*Prdm16*, *Pparg*, *Ucp1*), siRNAs (Scramble siRNA, si*Mettl3*-1, si*Mettl3*-2) and β-galactosidase (β-Gal) reporter plasmid by X-tremeGENE siRNA Transfection Reagent for 24 h. Cells were then induced to differentiate for 48 h. METTL3 and GAPDH protein levels were measured by immunoblotting **g**. Relative *Prdm16*, *Pparg,* and *Ucp1* luciferase activity were measured and normalized to β-Gal levels **h**–**j** (Scr in **h**, *n* = 4; others, *n* = 5 biologically independent cell samples for each group). Data represent the mean ± SEM. Significance was determined by unpaired two-tailed Student’s *t* test analysis. *p < 0.05. **p < 0.01. Source data are provided as a Source Data file.

To further determine whether METTL3 regulates m^6^A modification of mRNA related to brown fat differentiation and thermogenesis, we performed m^6^A RNA immunoprecipitation sequencing (m^6^ARIP-seq) analysis in iBAT of BKO and control mice. Each sample was pooled from five mice for each group. Consistent with published m^6^ARIP-seq results^41^, the m^6^A peaks identified in iBAT of flox/flox control mice were enriched at stop codon and 3′-UTR and were characterized by the canonical GGACU motif (Fig. 4a, b). However, the m^6^A peaks in iBAT of BKO mice were dispersed at transcription start site, 5′-UTR, and stop codon, and the enriched motif was CCAUG (Fig. 4a, b). In the iBAT of flox/flox mice, we identified about 9989 significant m^6^A peaks (false discovery rate < 0.05) in ~ 6845 transcripts (Fig. 4c and Supplementary Fig. 4). There were 5383 transcripts exhibiting decreased m^6^A levels in the iBAT of BKO mice (Fig. 4c and Supplementary Fig. 4). Importantly, m^6^A peaks in *Prdm16*, *Pparg* and *Ucp1* transcripts were decreased in the iBAT of BKO mice (Fig. 5a–c). Consistently, m^6^ARIP-RT-qPCR showed that the m^6^A modification in *Prdm16*, *Pparg,* and *Ucp1* transcripts were dramatically decreased in iBAT of BKO mice (Fig. 5d–f), which may contribute to reduced expression of *Prdm16*, *Pparg*, and *Ucp1*.

To further test whether the m^6^A modification at last exon and 3′-UTR of *Prdm16*, *Pparg,* and *Ucp1* transcripts were associated with the expression of *Prdm16*, *Pparg,* and *Ucp1*. We cloned the region into pMIR-REPORT Luciferase vector and performed luciferase assays in primary brown preadipocytes. Knockdown of *Mettl3* by two individual siRNAs (si*Mettl3*-1 or si*Mettl3*-2) in primary brown preadipocytes resulted in a significant reduction of METTL3 protein levels (Fig. 5g). Either si*Mettl3*-1 or si*Mettl3*-2 significantly decreased *Prdm16*, *Pparg,* and *Ucp1* luciferase activity, respectively (Fig. 5h–j). To identify which m^6^A readers were responsible for this regulation, we performed siRNA-mediated m^6^A reader proteins (Ythdf1, Ythdf2, or Ythdf3) knockdown and luciferase assays. As shown in Supplementary Fig. 5a–h, knockdown of *Ythdf1* by two individual siRNAs (si*Ythdf1*-1 or si*Ythdf1*-2) did not affect the luciferase activity of *Prdm16*, *Pparg,* or *Ucp1*, whereas knockdown of *Ythdf2* by two individual siRNAs (si*Ythdf2*-1 or si*Ythdf2*-2) significantly reduced their luciferase activity. In addition, knockdown of *Ythdf3* by si*Ythdf3*-2 but not si*Ythdf3*-1 significantly decreased *Prdm16* and *Ucp1* luciferase activity (Supplementary Fig. 5i–l). Moreover, the expression of *Ythdf2* and *Ythdf3* were significantly increased in mature adipocytes compared with preadipocytes (Supplementary Fig. 5m–o), indicating that YTHDF2 and YTHDF3 may regulate brown adipocyte differentiation and BAT development. These data also indicate that METTL3 and YTHDF2/3 may coordinate with each other and regulate BAT development.

### METTL3 is essential for brown adipogenesis in vitro

**Fig. 6:**
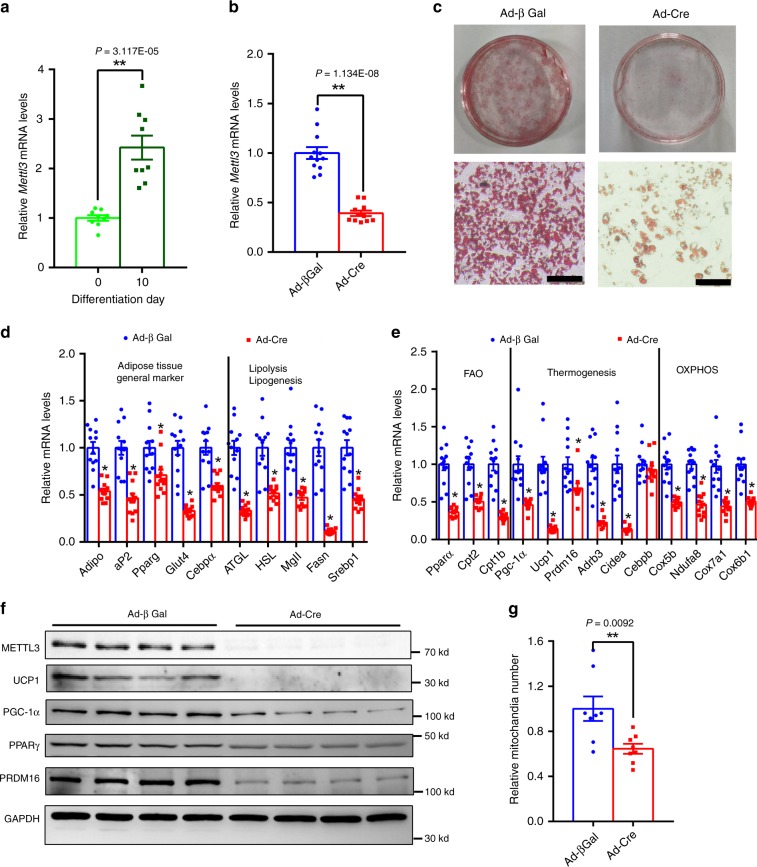
METTL3 is essential for brown adipogenesis. **a** The *Mettl3* mRNA levels in mature primary brown adipocytes (10 days after differentiation) and preadipocytes (before differentiation) (*n* = 9 for each group). **b** Relative *Mettl3* mRNA levels in primary brown adipocytes from *Mettl3*^flox/flox^ mice infected with Ad-Cre or Ad-βGal (Ad-Cre, *n* = 12; Ad-βGal, *n* = 11). **c** Oil red O staining of primary brown adipocytes from *Mettl3*^flox/flox^ mice infected with Ad-Cre or Ad-βGal. Scale bars represent 100 μm. **d**, **e** Real-time qPCR analysis of mRNA levels of genes involved in adipogenesis, lipolysis, lipogenesis, thermogenesis, fatty acid oxidation, thermogenesis and mitochondrial oxidative phosphorylation in primary brown adipocytes infected with Ad-Cre or Ad-βGal (*n* = 10–12 for each group). **f** Western blot analysis of protein levels of METTL3, UCP1, PGC-1α, PPARγ, PRDM16, and GAPDH in primary brown adipocytes described in **d** (*n* = 4 for each group). **g** Relative mitochondria number in primary brown adipocytes infected with Ad-Cre or Ad-βGal (*n* = 8 for each group). *n* was the number of biologically independent cell samples, and these cell culture experiments were repeated three times independently with similar results. Data represent the mean ± SEM. Significance was determined by unpaired two-tailed Student’s *t* test analysis. *p < 0.05. **p < 0.01. Source data are provided as a Source Data file.

*Mettl3* mRNA levels were significantly higher in mature primary brown adipocytes compared with preadipocytes (Fig. 6a), suggesting that METTL3 may directly regulate differentiation of brown adipocytes. To further determine whether METTL3 directly regulates the differentiation of brown adipocytes, we harvested the stromal–vascular fraction from the brown fat pads of *Mettl3*^flox/flox^ mice. Primary brown preadipocytes were infected with Ad-βGal and Ad-Cre adenovirus and differentiated to mature brown adipocytes. As expected, Cre adenovirus infection caused the deletion of *Mettl3* in primary brown adipocytes (Fig. 6b, f). Deletion of *Mettl3* significantly impaired the differentiation of precursor cells, as revealed by decreased Oil Red O staining (Fig. 6c) and the expression of general brown adipocyte markers (Fig. 6d). In addition, deletion of *Mettl3* in primary brown adipocytes significantly decreased the expression of genes involved in differentiation (*Prdm16* and *Pparg*), thermogenesis (*Pgc-1α*, *Ucp1*, *Adrb3*, and *Cidea*), lipogenesis (*Srebp1* and *Fasn*), lipolysis (*ATGL, HSL*, and *MgII*), fatty acid oxidation (*Ppara, Cpt2*, and *Cpt1b*) and oxidative phosphorylation (*Cox5b, Ndufa8, Cox7a1*, and *Cox6b1*) (Fig. 6d, e). UCP1, PGC-1α, PPARγ, and PRDM16 protein levels were also dramatically reduced in primary METTL3-deficient brown adipocytes (Fig. 6f). Consistent with decreased expression of PGC-1α, the relative mitochondria number was dramatically decreased by 33% in METTL3 knockout brown adipocytes (Fig. 6g). These results suggest that METTL3 is necessary for brown adipogenesis in vitro.

### BAT-specific Knockout of *Mettl3* results in decreased energy expenditure

**Fig. 7:**
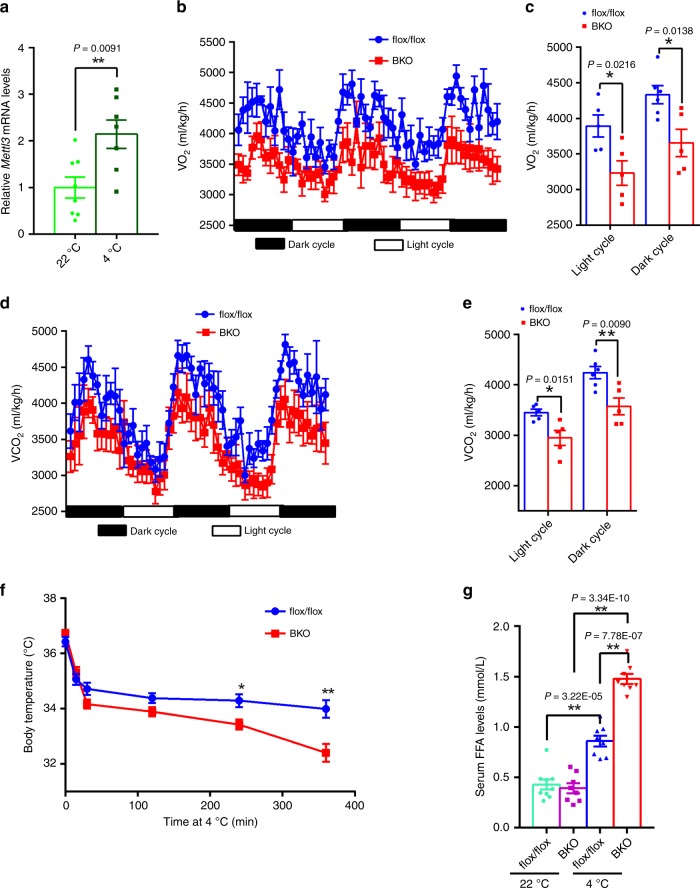
*Mettl3* deficiency in BAT decreases energy expenditure and impairs cold tolerance. **a** The *Mettl3* mRNA levels in iBATs of WT mice after acute cold exposure for 6 h (22 °C, *n* = 8; 4 °C; *n* = 7). **b**, **c** The O_2_ consumption rates in 8-week-old *Mettl3*^flox/flox^ and BKO mice at 22 °C (*n* = 5–6 for each group). **d**, **e** The CO_2_ production rates in 8-week-old *Mettl3*^flox/flox^ and BKO mice at 22 °C (*n* = 5–6 for each group). **f** The rectal temperature of 8-week-old *Mettl3*^flox/flox^ and BKO mice during acute cold exposure (4 °C) (*Mettl3*^flox/flox^, *n* = 10; BKO, *n* = 9). **g** Serum FFAs levels of 8-week-old *Mettl3*^flox/flox^ and BKO mice housed at room temperature or after cold exposure (4 °C) for 6 h (22 °C *Mettl3*^flox/flox^, *n* = 9; 22 °C BKO, *n* = 8; 4 °C *Mettl3*^flox/flox^, *n* = 8; 4 °C BKO, *n* = 8). Data represent the mean ± SEM. Significance was determined by unpaired two-tailed Student’s *t* test analysis. *p < 0.05. **p < 0.01. Source data are provided as a Source Data file.

Next, we tested the need for METTL3 in thermogenesis in vivo. *Mettl3* mRNA levels were significantly increased after acute cold exposure (4 °C for 6 h) (Fig. 7a), indicating that METTL3 may promote thermogenesis. BKO mice displayed lower oxygen (O_2_) consumption and CO_2_ production rates during both light and dark cycles (Fig. 7b–e), with similar amounts of food intake and physical activity compared with flox/flox controls (Supplementary Fig. 6a, b), indicating that energy expenditure was decreased in BKO mice. Furthermore, BKO mice were less able to defend against acute cold exposure (Fig. 7f). In addition, serum free fatty acid levels were much higher in BKO mice after cold challenge (Fig. 7g), which further supports impaired thermogenesis in BKO mice. The morphology of iBAT in BKO mice under the cold exposure condition still appeared abnormal, enlarged and “whitening” (Supplementary Fig. 7a–c). These data suggest that METTL3 in BAT is necessary for thermogenesis.

METTL3 was exclusively expressed in iBAT, and BAT-specific deletion of *Mettl3* impaired acute cold-induced thermogenesis, indicating that METTL3 may regulate browning of WAT in response to chronic cold exposure or the β-adrenergic agonist. To further test this hypothesis, BKO mice and flox/flox controls were exposed for chronic cold challenge (4 °C 7d) or injected with CL-316,243 for 4 days to induce browning of WAT. At room temperature, the weight of iWAT was similar between BKO and flox/flox mice (Supplementary Fig. 8a, c), and the thermogenesis related genes such as *Ucp1* and *Pgc-1α* were not changed in iWAT of BKO mice (Supplementary Fig. 8b, d). However, after either chronic cold exposure or multiple injection of CL-316,243, the weight of iWAT showed higher in BKO mice, and the expression of *Ucp1* and *Pgc-1α* were downregulated under both conditions (Supplementary Fig. 8a–d). These data indicate that BAT-specific deletion of *Mettl3* impairs the browning of WAT in response to chronic cold exposure or the β-adrenergic agonist.

### BAT-specific knockout of *Mettl3* predisposes to high-fat diet-induced obesity

**Fig. 8:**
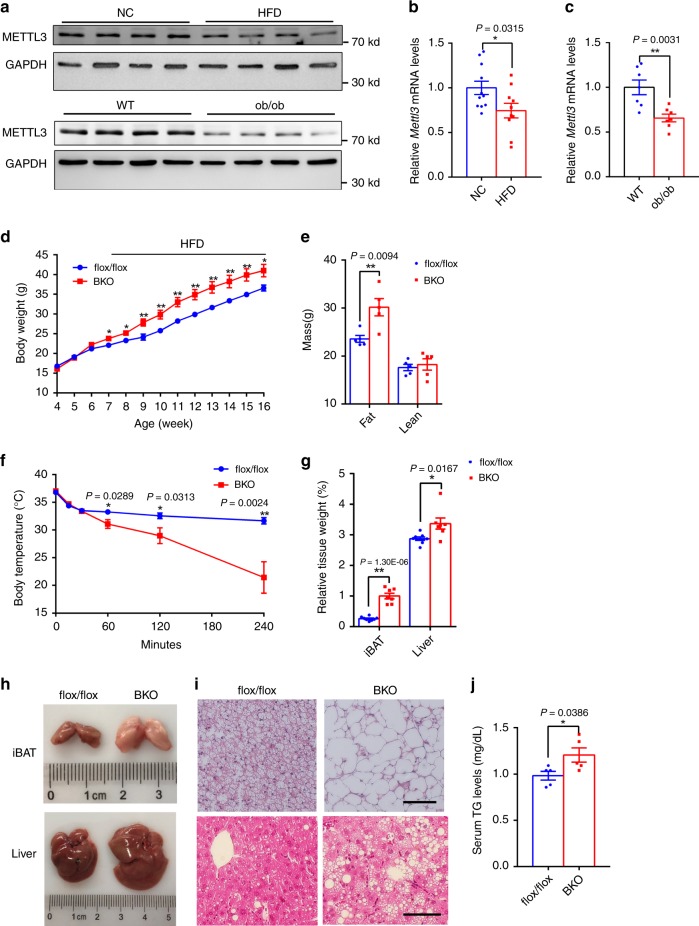
BAT-specific knockout of *Mettl3* predisposes to HFD-induced obesity. **a**–**c** The METTL3 protein and mRNA levels in iBATs of HFD-fed mice, ob/ob mice and their respective control mice (NC, *n* = 11; HFD, *n* = 10; WT, *n* = 7; ob/ob, *n* = 7). **d** The growth curve of METTL3 BKO and *Mettl3*^flox/flox^ mice fed HFD diet (5 weeks *Mettl3*^flox/flox^ and BKO, *n* = 10 for each group; 6 weeks *Mettl3*^flox/flox^, *n* = 10; 6 weeks BKO, *n* = 12; 7–16 weeks *Mettl3*^flox/flox^ and BKO, *n* = 12 for each group). **e** The fat and lean mass of 20-week-old HFD-fed mice (*n* = 5 for each group). **f** The rectal temperature of HFD-fed *Mettl3*^flox/flox^ and BKO mice during acute cold exposure (4 °C) (*Mettl3*^flox/flox^, *n* = 6; 0, 60, 120 min BKO, *n* = 5; 240 min BKO, *n* = 4). **g** The relative tissue weight of iBAT and liver in 20-week-old HFD-fed mice (*Mettl3*^flox/flox^, *n* = 8; BKO, *n* = 7). **h** Representative images of iBAT and liver from 20-week-old HFD-fed mice. **i** Hematoxylin and eosin (H&E) staining of iBAT and liver from 20-week-old HFD-fed mice. Scale bars represent 100 μm. Three mice for each group were used for H&E staining with similar results. **j** The serum TG levels of 20-week-old HFD-fed mice (*n* = 5 for each group). Data represent the mean ± SEM. Significance was determined by unpaired two-tailed Student’s *t* test analysis.*p < 0.05.**p < 0.01. Source data are provided as a Source Data file.

Reduced BAT thermogenesis contributes to obesity in both rodents and humans^42,43^. We next determined whether BAT *Mettl3* expression was associated with obesity by measuring BAT *Mettl3* expression in two obese mice models (high-fat diet-induced obese (DIO) and leptin-deficient ob/ob mice). METTL3 protein levels were lower in both DIO and ob/ob mice (Fig. 8a). *Mettl3* mRNA levels were also significantly reduced in both DIO and ob/ob mice (Fig. 8b, c). These data suggest that METTL3 in iBAT might regulate energy metabolism and obesity.

To determine whether BKO mice are sensitive to HFD-induced obesity, BKO and control mice were fed with HFD (45% fat), and body weight was measured weekly. As shown in Fig. 8d, BKO mice gained more body weight than flox/flox mice after feeding with HFD. Fat mass was significantly increased in BKO mice, whereas the lean mass did not change (Fig. 8e). BKO mice under a HFD feeding condition also showed severely impaired cold tolerance (Fig. 8f). The relative weight of iBAT and liver was much higher in BKO mice (Fig. 8g). Consistently, the size of iBAT and liver were larger in BKO mice (Fig. 8h). Furthermore, hematoxylin and eosin staining showed adipocyte hypertrophy and hepatosteatosis in BKO mice (Fig. 8i). Serum triglyceride levels were significantly increased in BKO mice (Fig. 8j). These data demonstrate that BKO mice were more prone to HFD-induced obesity.

**Fig. 9:**
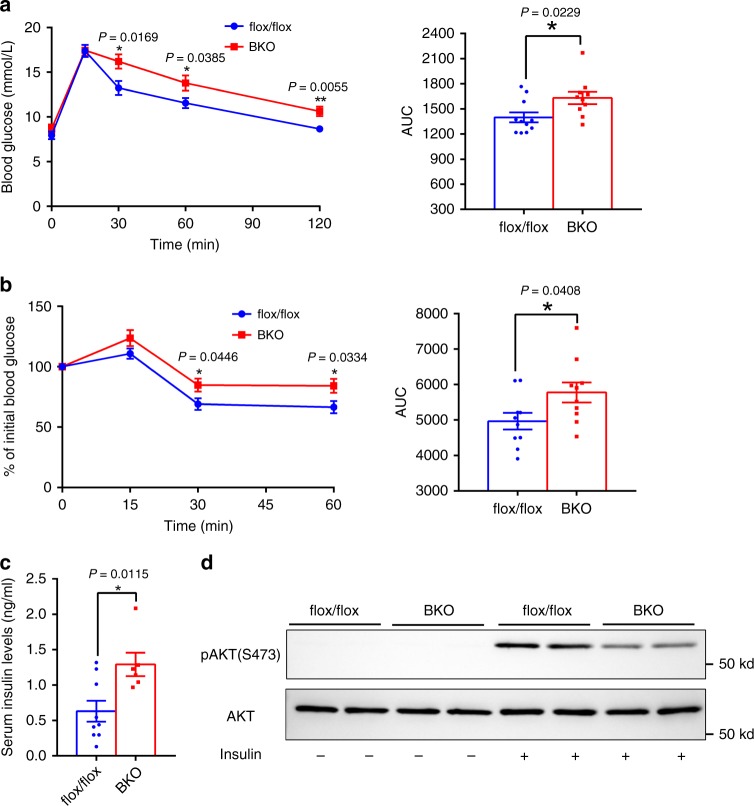
BAT-specific knockout of *Mettl3* predisposes to HFD-induced glucose intolerance and insulin resistance. **a** GTTs of 16-week-old HFD-fed mice (*Mettl3*^flox/flox^, *n* = 11; BKO, *n* = 10). **b** ITTs of 16-week-old HFD-fed mice (*n* = 10 for each group). **c** The serum insulin levels of 20-week-old HFD-fed mice (*Mettl3*^flox/flox^, *n* = 9; BKO, *n* = 6). **d** p-AKT(S473) and AKT protein levels of 20-week-old HFD-fed mice. This experiment was repeated three times by using three different groups of mouse samples with similar results. Data represent the mean±SEM. Significance was determined by unpaired two-tailed Student’s *t* test analysis. *p < 0.05. Source data are provided as a Source Data file.

Obesity leads to impaired glucose metabolism and insulin resistance. To determine whether BAT METTL3 regulates systemic glucose homeostasis and insulin resistance, we performed glucose tolerance tests and insulin tolerance tests on mice fed with HFD for 8 weeks (16 weeks old). As shown in Fig. 9a, b, BKO mice displayed glucose intolerance and insulin resistance. Serum insulin levels in BKO mice were also higher (Fig. 9c), further supporting that BKO mice showed insulin resistance. Moreover, insulin-induced p-AKT(S473) levels were significantly decreased in the livers of BKO mice (Fig. 9d). These data suggest that decreased expression of *Mettl3* in iBAT impairs systemic energy homeostasis, contributing to HFD-induced obesity and metabolic syndrome.

## Discussion

In recent years, much progress has been made in identifying transcriptional factors and cofactors in the regulation of brown cell differentiation and thermogenesis. However, whether mRNA m^6^A modification regulates iBAT development and thermogenesis is largely unknown. In this study, we demonstrated that METTL3 is an essential iBAT-enriched RNA methyltransferase and controls postnatal development of iBAT and thermogenesis by regulating m^6^A modification and expression of *Prdm16, Pparg,* and *Ucp1* transcripts.

METTL3 is selectively expressed in iBAT and associated with brown fat cell differentiation. Deletion of *Mettl3* leads to dramatically impaired brown adipocyte differentiation by suppressing brown fat related genes, including *Prdm16, Pparg, Pgc-1α,* and *Ucp1*. Consistent with the in vitro observations, BAT-specific *Mettl3* knockout mice display impaired postnatal development of iBAT and thermogenesis in vivo. iBAT in BKO mice displays steatotic hypertrophy and decreased mitochondria number at a very early age because of decreased expression of *Prdm16, Pparg, Pgc-1α*, and other BAT-selective genes. Therefore, BKO mice show reduced energy expenditure and are predisposed to HFD-induced obesity and metabolic syndrome.

A key question is how METTL3 regulates the postnatal development of a brown fat gene program. METTL3 is well known for RNA m^6^A modification. It has been reported that METTL3 regulates stem cell differentiation by promoting RNA m^6^A modification of key transcriptional factors^44^. METTL3 also has been linked to white fat cell differentiation^37^. BAT-specific deletion of *Mettl3* decreases iBAT-specific mRNA m^6^A modification and expression, including *Prdm16, Pparg,* and *Ucp1* transcripts.

One target of METTL3 is *Prdm16*, which has been shown to drive the differentiation of brow fat cells and browning of WAT by activating the expression of BAT-selective genes including *Ucp1* and *Pgc-1α*^11,45,46^. Similarly, BAT-specific deletion of *Mettl3* impaired BAT development and also impaired the development of beige adipocytes in iWAT. PRDM16 also controls the myoblast/brown fat switch^47^. Knockdown of *Prdm16* downregulates BAT-selective genes and upregulates skeletal muscle-associated genes^11,47^. Likewise, BAT-specific deletion of *Mettl3* also downregulates BAT-selective genes and upregulates skeletal muscle-associated genes, which is likely owing to decreased PRDM16 expression. Mechanistically, BAT-specific deletion of *Mettl3* decreases m^6^A peaks of the *Prdm16* transcript, which may be associated with decreased *Prdm16* mRNA levels with the involvement of YTHDF2/3. These data indicate that METTL3 regulates iBAT postnatal development at least partially by regulating m^6^A modification and expression of the *Prdm16* transcript. However, BAT-specific *Prdm16* knockout mice show normal postnatal development of iBAT at a young age and display “whitening” and enlarged iBAT in old age^10^, whereas *Mettl3* BKO mice show severely impaired postnatal development of iBAT at 5 days of age, suggesting that METTL3 in iBAT has certain actions that probably do not occur solely through modulation of *Prdm16* expression.

Another target of METTL3 is *Pparg*, which has an essential role in adipocyte differentiation. *Pparg*-deficient mice display no brown or white adipose tissues^48,49^, suggesting that PPARγ is necessary for development of both BAT and white adipose tissue. BKO mice show significantly decreased m^6^A modification and expression of the *Pparg* transcript, which contributes to the impaired postnatal development of iBAT.

Another important target of METTL3 is *Ucp1*, which is a key thermogenic factor in iBAT. During postnatal development of iBAT, UCP1 and METTL3 protein levels are significantly increased. BAT-specific deletion of *Mettl3* decreases m^6^A peaks and expression of the *Ucp1* transcript, which contributes to impaired thermogenesis. However, *Ucp1* knockout mice do not show enlarged iBAT^50^, suggesting that BAT-specific deletion of *Mettl3* is less likely to result in enlarged iBAT solely through modulation of *Ucp1* expression. Thus, it is possible that METTL3 acts as an essential regulator of postnatal development of iBAT and thermogenesis at least partially by controlling m^6^A modification and expression of *Prdm16, Pparg,* and *Ucp1* transcripts simultaneously. However, we cannot rule out other possible mechanisms because METTL3 regulates m^6^A modification and expression of many other BAT-selective genes, based on the m^6^ARIP-seq and RNA-seq data.

METTL3-mediated mRNA m^6^A modification can be recognized by the individual m^6^A reader proteins, which play key roles in controlling gene expression. Early studies showed that YTHDF1 binding promotes translational efficiency^51^, YTHDF2-binding increases mRNA decay^19^, and YTHDF3 can assist the effects of YTHDF1 and YTHDF2^52,53^. However, other roles for these reader proteins are rapidly emerging in the regulation of specific mRNAs in different types of cells during different stimuli^29,54,55^. In addition, the profiles and function of METTL3-mediated mRNA m^6^A are different depending on tissue and developmental stage^41,56,57^. For example, hypoxia increases m^6^A content of mRNA and enhances the stability of m^6^A modified mRNAs^24^, raising the possibility that m^6^A modification could also stabilize transcripts. In iSLK.219 cells, both METTL3 and YTHDF2 appear to promote lytic viral gene expression during Kaposi’s sarcoma-associated herpesvirus infection^58^. Another study shows that YTHDF2 also recognizes multiple m^6^A sites in the SV40 late transcripts and strongly promotes SV40 replication^59^. Our study shows that the expression of *Mettl3* and *Ythdf2* are increased during brown adipocyte differentiation. BAT-specific deletion of *Mettl3* reduces m^6^A modification and expression of *Prdm16, Pparg,* and *Ucp1* transcripts, indicating that m^6^A modification is required for the expression of *Prdm16, Pparg,* and *Ucp1* transcripts in iBAT. Knockdown of either *Mettl3* or *Ythdf2* decreases the luciferase activity of *Prdm16, Pparg,* and *Ucp1* in primary brown preadipocytes, indicating that METTL3 and YTHDF2 may play a similar role in brown adipogenesis. Future studies should clarify the function and the molecular mechanisms of YTHDF2 in the regulation of BAT development.

In addition, m^6^A pathway has been shown to regulate white adipocyte differentiation^32,33,37,60,61^. The well-studied protein in m^6^A pathway is FTO, which was previously identified by human genome-wide associated studies with strong association with obesity^62^. FTO promotes white adipocyte differentiation in vitro and development of obesity in vivo^32,61^, whereas deletion of FTO impairs white fat adipogenesis both in vitro and in vivo^32,33,60,63^. The potential molecular targets include RUNX1T1, ATG5/7, and JAK2^32,33,60,61^. Interestingly, knockdown of m^6^A writer proteins (METTL3, METTL14, or WTAP) in 3T3-L1 cells results in cell cycle arrest and impaired adipogenesis by suppressing expression of cyclin A2^37^. These results indicate that m^6^A eraser and writer proteins play similar roles in white fat adipogenesis although their targets are different. With respect to WAT browning, FTO deficiency has been shown to induce UCP1 expression in white adipocytes^64^, whereas our study shows that BAT-specific deletion of *Mettl3* impairs the browning of WAT in response to chronic cold exposure or the β-adrenergic agonist, indicating that m^6^A eraser and writer proteins may play opposite roles in regulation of WAT browning and BAT development. Further investigations are necessary to fully explore the mechanisms of RNA m^6^A pathway (including other writer, reader, and eraser proteins) in controlling postnatal development of iBAT and energy metabolism.

In conclusion, we have shown that METTL3 is an essential BAT-enriched RNA methyltransferase and controls postnatal development of iBAT by regulating m^6^A modification and expression of *Prdm16, Pparg,* and *Ucp1* transcripts. BAT-specific knockout of *Mettl3* leads to a marked reduction of BAT-mediated adaptive thermogenesis and results in obesity and systemic insulin resistance. These data demonstrate that METTL3 is an essential regulator that controls postnatal development of iBAT and energy homeostasis.

## Methods

### Animal experiments

Animal experiments were carried out in strict accordance with the Guide for the Care and Use of Laboratory Animals published by the US National Institutes of Health (NIH publication no. 85–23, revised 1996) and approved by the Institutional Animal Care and Use Committee or Animal Experimental Ethics Committee of Harbin Institute of Technology (HIT/IACUC). The permit number is IACUC-2018002. Mice were housed under controlled light (12-h light/12-h dark cycle), temperature (24 ± 2 °C) and humidity (50 ± 10%) conditions and fed a normal chow diet with a free access to water. For diet-induced obesity studies, mice were fed with an HFD (MD12032, 45% fat, Medicience). *Mettl3*^flox/flox^ mice, in which the exon 2 and exon 3 of *Mettl3* gene was flanked by two loxp sites, were generated by using CRISPR-Cas9 technique (Supplementary Fig. 1A). Southern blot data showed that the homologous recombination in *Mettl3*^flox/+^ mice were correct (Supplementary Fig. 1B). *Ucp1*-iCre mice, in which IRES-Cre was inserted between exon 6 and the 3′-UTR to allow *Ucp1* and iCRE expression at the same time with lower levels, have been shown previously^38^. BAT-specific *Mettl3* knockout mice were generated by crossing *Mettl3* ^flox/flox^ mice with *Ucp1*-iCre mice.

### Body composition and energy metabolism measurement

Body weight was measured weekly. Body composition (fat and lean mass) was determined by a body composition analyzer (Brucker Minispec LF50). For metabolic studies, mice were housed individually in metabolic cages (Promethion, Sable Systems, LasVegas, NV), and free access to food and water. Oxygen consumption and CO_2_ production rates were monitored for 72 hr. Food intake and physical activity monitoring were performed simultaneously with metabolic measurements. Data were collected and analyzed by MetaScreen-Data Collection Software (V2.3.15) and Expedata-P Data Analysis Software (V1.9.17), respectively.

### Glucose tolerance tests and Insulin tolerance tests

For glucose tolerance test experiment, mice fasted for 6 hr were injected intraperitoneally with d-glucose (1 g/kg). For insulin tolerance test experiment, mice fasted for 6 h were injected intraperitoneally with human insulin (Lily) (1 U/kg). Blood glucose levels were measured from the tail vein at indicated times using a glucometer^65^. Blood samples were collected from orbital sinus. Serum insulin levels were measured using insulin ELISA kits (MS100, EZassay).

### In vivo insulin stimulation assay

20-week-old HFD-fed *Mettl3* ^flox/flox^ and BKO mice were fasted for 20–24 h, anesthetized, and administrated insulin (2 units/kg body weight) via inferior vena. Livers were isolated and homogenized in a lysis buffer (R0020, Solarbio). Liver extracts were immunoblotted with antibodies against phospho-AKT (pSer473 from Cell Signaling Technology) and AKT (Cell Signaling Technology).

### Cold-stress experiments

For cold exposure experiments, individual mouse was placed in a single cage in a cold room (4 °C) with free access to water. Control mice were kept at room temperature (22 °C). The core body temperature was monitored using a rectal probe (7001HT, Phyritemp) at each time point. For chronic cold exposure, mice were first treated with 3 days of the intermittent cold exposure (12 h at 4 °C in the light cycle and 12 h at room temperature), and then followed by 4 days of continued cold exposure at 4 °C^66,67^.

### Chronic CL-316,243 treatment

BKO and flox/flox mice were injected intraperitoneally with CL-316,243 at 1 mg/kg body weight or equal volume of saline daily for 4 days. Mice were killed on day 5 without addition injection.

### Immunoblotting

Cells or tissues were homogenized in an l-RIPA lysis buffer (R0020, Solarbio). Protein was separated by sodium dodecyl sulfate–polyacrylamide gel electrophoresis, immunoblotted with the indicated antibodies, and visualized using the ECL. The antibody information and dilutions were as follows: METTL3(D2160,Cell Signaling Technology),1:3000; Akt(9272, Cell Signaling Technology),1:5000; p-Akt (S473)(9271, Cell Signaling Technology),1:5000; UCP1(U6382, Sigma),1:5000; PGC-1a(66369-1-lg, Proteintech),1:1000; β-Actin(60008-1-lg, Proteintech),1:5000; PPARg(16643-1-AP, Proteintech),1:300 0; GAPDH(60004-1-lg, Proteintech),1:5000; Total OXPHOS (ab110413, Abcom),1:2000; PRDM16(A11581, ABclonal),1:1000; YTHDF1(17479-1-AP, proteintech),1:2000; YTHDF2(24744-1-AP, proteintech),1:5000; YTHDF3(25537-1-AP, proteintech),1:3000.

### Quantitative Real-Time PCR (qPCR)

Total RNA was isolated using TriPure Isolation Reagent (94015120, Roche), and the first-strand cDNAs were synthesized using Random Primers and M-MLV reverse transcriptase (M1701, Promega)^65,68^. RNA abundance was measured using SYBR Green Mixs (4913914001, Roche) and Roche LightCycler 480 real-time PCR system (Roche, Mannheim, Germany). The expression of individual genes was normalized to the expression of 36B4, a house-keeping gene. Primers for real-time qRT-PCR were listed in Supplementary Table 1.

### Quantification of mtDNA copy number

Total DNA was isolated from mouse brown adipose tissue. mtDNA was amplified using primers specific for the mitochondrial mtND1 gene and normalized to genomic DNA by amplification of the LPL nuclear gene. The primer sequences can be found in Supplementary Table 1.

### Primary brown adipocytes culture and adenovirus infection

The interscapular brown fat pad was dissected from 4–5-week-old *Mettl3*^flox/flox^ or C57BL/6 wildtype mice, minced, and then digested for 20–30 min at 37 °C in PBS containing 10 mm CaCl2, 1.5 mg/ml Collagenase type II, and 1.4 U/ml Dispase II. Digested tissue was filtered through a 100 µm cell strainer to remove large pieces, and then centrifuged for 10 min at 1000 *g* to pellet the stromal–vascular fraction (SVF) cells. SVF cells were resuspended in complete culture medium (DMEM with 10% FBS and Pen/Strep), and then plated on collagen-coated 24-well plates. For preadipocyte differentiation, cells grown to 100% confluence (Day 0) were exposed to induction in DMEM containing 2 μg/mL dexamethasone, 1 μm insulin, 0.5 mm isobutylmethylxanthine, 1 μm rosiglitazone, 1 nm T3, 62.5 μm indomethacin, and 10% FBS. Three days after induction (from Day 3), cells were maintained in media containing 1 μm insulin, 1 nm T3 and 10% FBS until ready for harvest (generally day 6–7 post differentiation). All chemicals for cell culture were obtained from Sigma-Aldrich. For adenoviral infection of primary SVF cells, 100% confluent cells were infected with Cyclization Recombination Enzyme (Cre) or βGal-expressing adenovirus in growth medium overnight. The medium was then switched to induction medium for 72 hr to induce adipogenic differentiation, and then cells were maintained in differentiation medium until ready for harvest. For oil-red staining, infected cells were maintained in differentiation medium for 3 days. For RT-qPCR and Western blot, infected cells were maintained in differentiation medium for 1 day.

### RNA-seq and m^6^ARIP-seq

Total RNA was extracted using Tripure Isolation Reagent (94015120, Roche) from iBAT of *Mettl3*^flox/flox^ and BKO mice at 8 weeks old. Each sample was pooled from four mice for each group. RNA-seq was performed by using Illumina NovaSeq 6000 platform. Paired-end clean reads were aligned to the mouse reference genome (Ensemble_GRCm38.90) with TopHat (version 2.0.12), and the aligned reads were used to quantify mRNA expression by using HTSeq-count (version 0.6.1).

For m^6^ARIP-seq, total RNA was extracted using Tripure Isolation Reagent (94015120, Roche) from iBAT of *Mettl3*^flox/flox^ and BKO mice at 8 weeks old. Each sample (300 μg total RNA) was pooled from five mice for each group. Poly(A)^+^ RNA was purified using Dynabeads mRNA Purification Kit (61006, Invitrogen) following the manufacturer’s instructions. Chemically fragmented poly(A)^+^ RNA was incubated with m^6^A antibody (202003, Synaptic System) for immunoprecipitation following the standard protocol of Magna MeRIP^TM^ m^6^A Kit (17-10499, MERK). Enrichment of m^6^A mRNA was then analyzed by high-throughput sequencing using Illumina Hiseq X platform. Sequenced reads were trimmed for adaptor sequence by using skewer (Version: 0.1.126), and then mapped to Ensemble_GRCm38.90 whole genome using BWA (Version: 0.7.12-r1039) with parameters -T 25; -k 18. The m^6^A peaks were detected by MACS2 (Version 2.1.0), and the motif search was detected by HOMER findMotifGenome.pl (Version: v4.9.1)^69^.

### Transfection, siRNA knockdown, and luciferase assays

Based on the above m^6^ARIP-seq data, we identified the last exon and 3′-UTR regions of *Prdm16* (4: 154318508- 154319005), *Pparg* (6: 115489946-115490374), and *Ucp1* (8: 83297793- 83298478), containing m^6^A modification sites. We cloned these regions into pMIR-REPORT Luciferase vector, respectively. Primary brown preadipocytes seeded in 24-well plates were co-transfected with these pMIR-REPORT Luciferase vectors, siRNAs (100 nm) and β-galactosidase (β-Gal) reporter plasmid by X-tremeGENE siRNA Transfection Reagent (Roche) for 24 h. Cells were then induced to differentiate for 48 h. Luciferase activity was measured using a luciferase assay system (Promega) and normalized to β-Gal levels. siRNA targeted sequences were as follows: si*Mettl3*-1: 5′-GCUACCGUAUGGGACAUUA-3′; si*Mettl3*-2: 5′-CGGCUAAGAAGUCAAGGAA-3′; si*Ythdf1*-1: 5′-GGGUUGAUUGUUGCAUCUUUA-3′; si*Ythdf1*-2: 5′-GCCCACAGCUAUAACCCUAAA-3′; si*Ythdf2*-1: 5′-GCAAACUUGCAGUUUAUGUAU-3′; si*Ythdf2*-2: 5′-CCAUGCCCUAUCUAACUUCUU-3′; si*Ythdf3*-1: 5′-ACCAAUGUCAGAUCCAUAUAU-3′; si*Ythdf3*-2: 5′-CGUGUGGAGAUGUCCUAUUAA-3′; Scramble siRNA: UUCUCCGAACGUGUCACGUTT.

### Statistical analysis

Data were presented as means ± S.E. Differences between groups were analyzed by unpaired two-tailed Student’s *t* tests. *p* < 0.05 was considered statistically significant. *p < 0.05. **p < 0.01.

### Reporting summary

Further information on research design is available in the Nature Research Reporting Summary linked to this article.

## Supplementary information

Supplementary Information

Reporting Summary

## Data Availability

The data supporting the findings are available within the article and Supplementary Information. RNA-seq data files have been deposited into Gene Expression Omnibus database (www.ncbi.nlm.nih.gov/geo) with accession number GSE133216. m^6^ARIP-seq data that support the findings of this study have been deposited in GEO under accession code GSE141076. The source data underlying Figs. 1a–b, 2a–f, 3c–f, 5d–j, 6a–j, 7a–g, 8a–j, and 9a–d and Supplementary Figs. 1b–d, 2a–d, 3a, 5a–o, 6a–b, 7a–c, and 8a–d are provided as a Source Data file. All other data are available from the authors upon request.

## Source data

Source Data

## References

[1] Wu J (2012). Beige adipocytes are a distinct type of thermogenic fat cell in mouse and human. Cell.

[2] Petrovic N (2010). Chronic peroxisome proliferator-activated receptor γ (PPARγ) activation of epididymally derived white adipocyte cultures reveals a population of thermogenically competent, UCP1-containing adipocytes molecularly distinct from classic brown adipocytes. J. Biol. Chem..

[3] Barbatelli G (2010). The emergence of cold-induced brown adipocytes in mouse white fat depots is determined predominantly by white to brown adipocyte transdifferentiation. Am. J. Physiol. Endocrinol. Metab..

[4] Lowell BB, Spiegelman BM (2000). Towards a molecular understanding of adaptive thermogenesis. Nature.

[5] Cypess AM (2009). Identification and importance of brown adipose tissue in adult humans. N. Engl. J. Med..

[6] Saito M (2009). High incidence of metabolically active brown adipose tissue in healthy adult humans: incidence of metabolically active brown adipose tissue in healthy adult human. Diabetes.

[7] Virtanen KA (2009). Functional brown adipose tissue in healthy adults. N. Engl. J. Med..

[8] Yoneshiro T (2013). Recruited brown adipose tissue as an antiobesity agent in humans. J. Clin. Investig..

[9] Xue B (2007). Genetic variability affects the development of brown adipocytes in white fat but not in interscapular brown fat. J. Lipid Res..

[10] Harms MJ (2014). Prdm16 is required for the maintenance of brown adipocyte identity and function in adult mice. Cell Metab..

[11] Seale P (2007). Transcriptional control of brown fat determination by PRDM16. Cell Metab..

[12] Harms MJ (2015). PRDM16 binds MED1 and controls chromatin architecture to determine a brown fat transcriptional program. Genes Dev..

[13] Lefterova MI, Lazar MA (2009). New developments in adipogenesis. Trends Endocrinol. Metab..

[14] Puigserver P (1998). A cold-inducible coactivator of nuclear receptors linked to adaptive thermogenesis. Cell.

[15] Inagaki T, Sakai J, Kajimura S (2016). Transcriptional and epigenetic control of brown and beige adipose cell fate and function. Nat. Rev. Mol. Cell Biol..

[16] Yue Y, Liu J, He C (2015). RNA N6-methyladenosine methylation in post-transcriptional gene expression regulation. Genes Dev..

[17] Liu J (2014). A METTL3-METTL14 complex mediates mammalian nuclear RNA N6-adenosine methylation. Nat. Chem. Biol..

[18] Ping XL (2014). Mammalian WTAP is a regulatory subunit of the RNA N6-methyladenosine methyltransferase. Cell Res..

[19] Wang X (2014). N6-methyladenosine-dependent regulation of messenger RNA stability. Nature.

[20] Zhang Z (2010). The YTH domain is a novel RNA binding domain. J. Biol. Chem..

[21] Jia G (2011). N6-methyladenosine in nuclear RNA is a major substrate of the obesity-associated FTO. Nat. Chem. Biol..

[22] Zheng G (2013). ALKBH5 is a mammalian RNA demethylase that impacts RNA metabolism and mouse fertility. Mol. Cell.

[23] Bartosovic M (2017). N6-methyladenosine demethylase FTO targets pre-mRNAs and regulates alternative splicing and 3’-end processing. Nucleic Acids Res..

[24] Fry NJ, Law BA, Ilkayeva OR, Holley CL, Mansfield KD (2017). N(6)-methyladenosine is required for the hypoxic stabilization of specific mRNAs. RNA.

[25] Choe J (2018). mRNA circularization by METTL3–eIF3h enhances translation and promotes oncogenesis. Nature.

[26] Alarcon CR, Lee H, Goodarzi H, Halberg N, Tavazoie SF (2015). N6-methyladenosine marks primary microRNAs for processing. Nature.

[27] Patil DP (2016). m(6)A RNA methylation promotes XIST-mediated transcriptional repression. Nature.

[28] Fustin JM (2013). RNA-methylation-dependent RNA processing controls the speed of the circadian clock. Cell.

[29] Xiang Y (2017). RNA m(6)A methylation regulates the ultraviolet-induced DNA damage response. Nature.

[30] Chen T (2015). m(6)A RNA methylation is regulated by microRNAs and promotes reprogramming to pluripotency. Cell Stem Cell.

[31] Geula S (2015). Stem cells. m6A mRNA methylation facilitates resolution of naive pluripotency toward differentiation. Science.

[32] Wang, X. et al. m6A mRNA methylation controls autophagy and adipogenesis by targeting Atg5 and Atg7. *Autophagy* 1–15 (2019). 10.1080/15548627.2019.1659617 PMC7469583 31451060

[33] Wu R (2019). m6A methylation modulates adipogenesis through JAK2-STAT3-C/EBPβ signaling. Biochim. Biophys. Acta.

[34] Ma C (2018). RNA m(6)A methylation participates in regulation of postnatal development of the mouse cerebellum. Genome Biol..

[35] Lin Z (2017). Mettl3-/Mettl14-mediated mRNA N(6)-methyladenosine modulates murine spermatogenesis. Cell Res..

[36] Xu K (2017). Mettl3-mediated m(6)A regulates spermatogonial differentiation and meiosis initiation. Cell Res..

[37] Kobayashi, M. et al. TheRNA methyltransferase complex of WTAP, METTL3, and METTL14 regulates mitotic clonal expansion in adipogenesis. *Mol. Cell Biol.* **38**, pii: e00116-18 (2018). 10.1128/MCB.00116-18 PMC6066751 29866655

[38] Li L (2017). Brown adipocytes can display a mammary basal myoepithelial cell phenotype in vivo. Mol. Metab..

[39] Bartelt A (2011). Brown adipose tissue activity controls triglyceride clearance. Nat. Med..

[40] Heine M (2018). Lipolysis triggers a systemic insulin response essential for efficient energy replenishment of activated brown adipose tissue in mice. Cell Metab..

[41] Dominissini D (2012). Topology of the human and mouse m6A RNA methylomes revealed by m6A-seq. Nature.

[42] Tokuyama K, Himms-Hagen J (1986). Brown adipose tissue thermogenesis, torpor, and obesity of glutamate-treated mice. Am. J. Physiol. Endocrinol. Metab..

[43] Vijgen GHEJ (2011). Brown adipose tissue in morbidly obese subjects. PLOS ONE.

[44] Lin S, Gregory RI (2014). Methyltransferases modulate RNA stability in embryonic stem cells. Nat. Cell Biol..

[45] Kajimura S (2015). Promoting brown and beige adipocyte biogenesis through the PRDM16 pathway. Int J. Obes..

[46] Seale P (2011). Prdm16 determines the thermogenic program of subcutaneous white adipose tissue in mice. J. Clin. Investig..

[47] Seale P (2008). PRDM16 controls a brown fat/skeletal muscle switch. Nature.

[48] Barak Y (1999). PPAR gamma is required for placental, cardiac, and adipose tissue development. Mol. Cell.

[49] Rosen ED (1999). PPAR gamma is required for the differentiation of adipose tissue in vivo and in vitro. Mol. Cell.

[50] Enerback S (1997). Mice lacking mitochondrial uncoupling protein are cold-sensitive but not obese. Nature.

[51] Wang X (2015). N(6)-methyladenosine modulates messenger RNA translation efficiency. Cell.

[52] Li A (2017). Cytoplasmic m(6)A reader YTHDF3 promotes mRNA translation. Cell Res.

[53] Shi H (2017). YTHDF3 facilitates translation and decay of N(6)-methyladenosine-modified RNA. Cell Res.

[54] Meyer KD (2015). 5’ UTR m(6)A promotes cap-independent translation. Cell.

[55] Yu J, Li Y, Wang T, Zhong X (2018). Modification of N6-methyladenosine RNA methylation on heat shock protein expression. PLoS ONE.

[56] Wu R, Jiang D, Wang Y, Wang X (2016). N (6)-methyladenosine (m(6)A) methylation in mRNA with a dynamic and reversible epigenetic modification. Mol. Biotechnol..

[57] Wu Y (2018). Mettl3-mediated m(6)A RNA methylation regulates the fate of bone marrow mesenchymal stem cells and osteoporosis. Nat. Commun..

[58] Hesser CR, Karijolich J, Dominissini D, He C, Glaunsinger BA (2018). N6-methyladenosine modification and the YTHDF2 reader protein play cell type specific roles in lytic viral gene expression during Kaposi’s sarcoma-associated herpesvirus infection. PLoS Pathog..

[59] Tsai K, Courtney DG, Cullen BR (2018). Addition of m6A to SV40 late mRNAs enhances viral structural gene expression and replication. PLoS Pathog..

[60] Zhao X (2014). FTO-dependent demethylation of N6-methyladenosine regulates mRNA splicing and is required for adipogenesis. Cell Res..

[61] Merkestein M (2015). FTO influences adipogenesis by regulating mitotic clonal expansion. Nat. Commun..

[62] Loos RJF, Yeo GSH (2014). The bigger picture of FTO—the first GWAS-identified obesity gene. Nat. Rev. Endocrinol..

[63] Fischer J (2009). Inactivation of the Fto gene protects from obesity. Nature.

[64] Tews D (2013). FTO deficiency induces UCP-1 expression and mitochondrial uncoupling in adipocytes. Endocrinology.

[65] Ren X (2017). A small-molecule inhibitor of NF-κB-inducing kinase (NIK) protects liver from toxin-induced inflammation, oxidative stress, and injury. FASEB J..

[66] Bartelt A (2017). Thermogenic adipocytes promote HDL turnover and reverse cholesterol transport. Nat. Commun..

[67] Shin H (2017). Lipolysis in brown adipocytes is not essential for cold-induced thermogenesis in mice. Cell Metab..

[68] Li X (2018). Islet α-cell inflammation induced by NF-κB inducing kinase (NIK) leads to hypoglycemia, pancreatitis, growth retardation, and postnatal death in mice. Theranostics.

[69] Dominissini D, Moshitch-Moshkovitz S, Salmon-Divon M, Amariglio N, Rechavi G (2013). Transcriptome-wide mapping of N(6)-methyladenosine by m(6)A-seq based on immunocapturing and massively parallel sequencing. Nat. Protoc..

