# Supplementary material for: METTL3 is essential for postnatal development of brown adipose tissue and energy expenditure in mice
Source: Nat Commun. 2020 Apr 3;11:1648. doi: 10.1038/s41467-020-15488-2 (PMC7125133; doi:10.1038/s41467-020-15488-2)
Supplement: Supplementary file 1 — Supplementary Information [file 41467_2020_15488_MOESM1_ESM.pdf]

**METTL3 is essential for postnatal development of brown adipose tissue and energy expenditure in mice**

**Wang et al.**

# Supplementary Figure 1

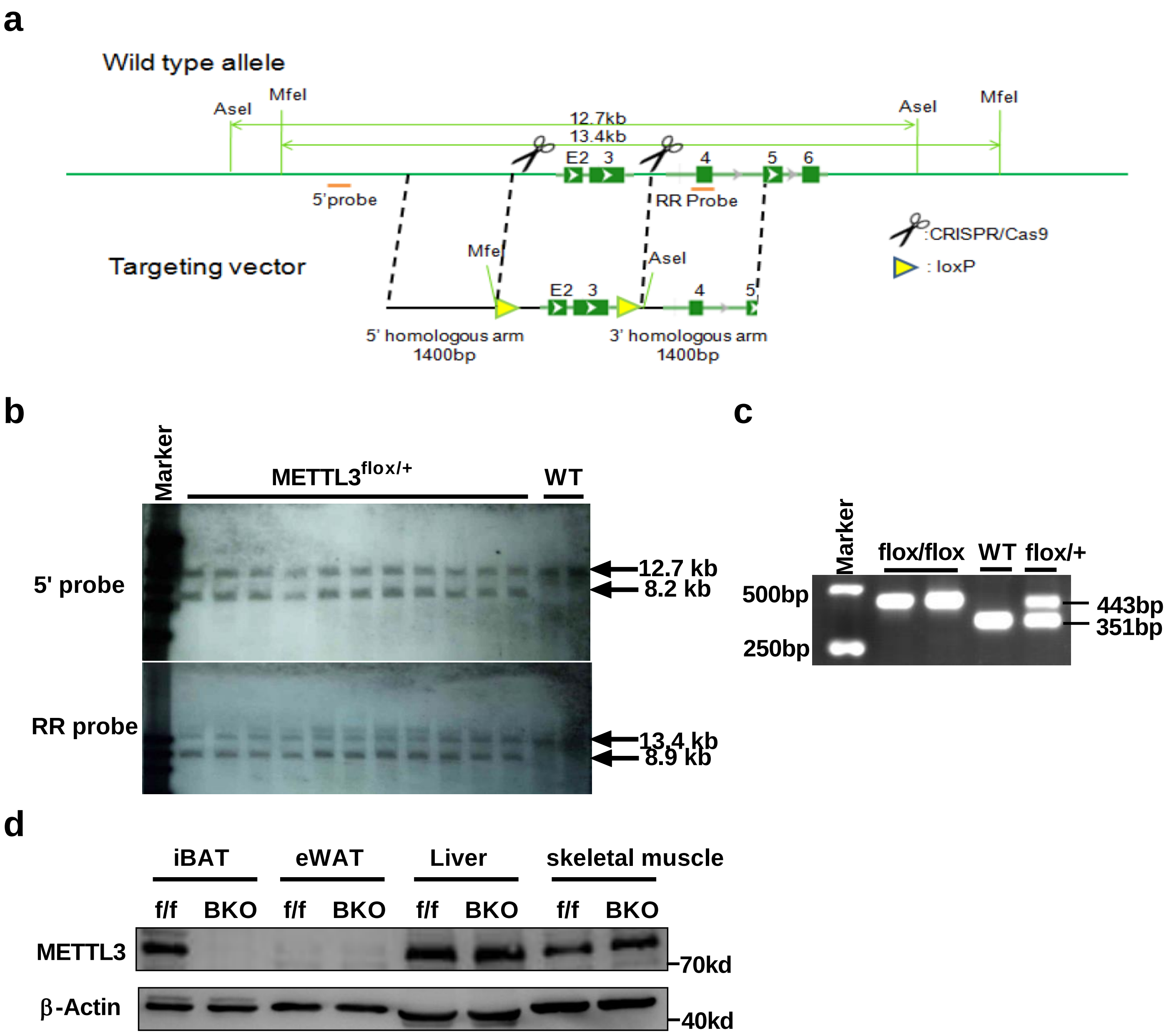


**Supplementary Figure 1. Generation of *Mettl3*$^{flox/flox}$ and *Mettl3* BKO mice.**

(a) *Mettl3* alleles and targeting construct. Top: wildtype *Mettl3* allele (exons indicated as solid green rectangles). Two flanking DNA fragments used for Southern hybridization are shown in red. Middle: targeting vector containing two loxP sites (yellow triangles). Insertion of the targeting vector by CRISPR/Cas9 technique yielded the *Mettl3* lox allele. (b) Southern blot of tail genome DNA from *Mettl3*$^{flox/+}$ (heterozygous) and WT mice. (c) Genotyping of PCR products of *Mettl3*$^{flox/flox}$ (homozygous), *Mettl3*$^{flox/+}$ (heterozygous) and WT mice. (d)

METTL3 protein levels in iBAT, eWAT, liver and skeletal muscle of 8-week-old *Mettl3*$^{flox/flox}$ and BKO mice. These experiments were repeated at least three times independently with similar results.

Source data are provided as a Source Data file.

## Supplementary Figure 2

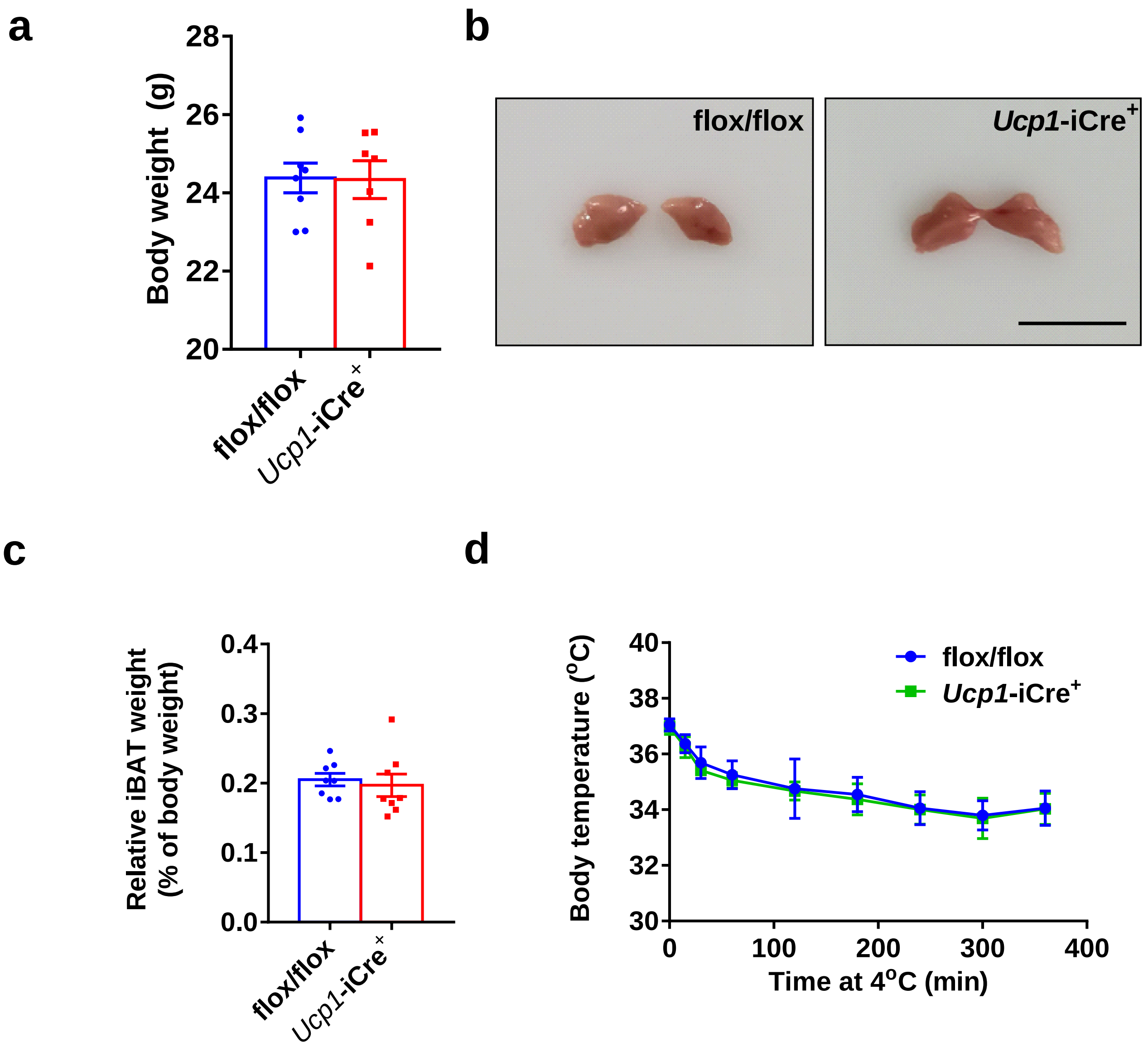


**Supplementary Figure 2.** ***Mettl3*$^{flox/flox}$ mice display similar body weight, similar size of iBAT and similar cold tolerance with *Ucp1*-iCre mice.**

(a)The body weight of 8-week-old *Mettl3*$^{flox/flox}$ and *Ucp1*-iCre mice (*Mettl3*$^{flox/flox}$, n = 8; *Ucp1*-iCre, n = 7).

(b, c) Gross appearance and relative weight of iBAT in *Mettl3*$^{flox/flox}$ and *Ucp1*-iCre mice at 8-week old (n=8 for each group). The scale bar represents 1 cm.

(d) The rectal temperature of 8-week-old *Mettl3*$^{flox/flox}$ and *Ucp1*-iCre mice during acute cold exposure (4℃) (n=7 for each group).

Data represent the mean ± SEM. Source data are provided as a Source Data file.

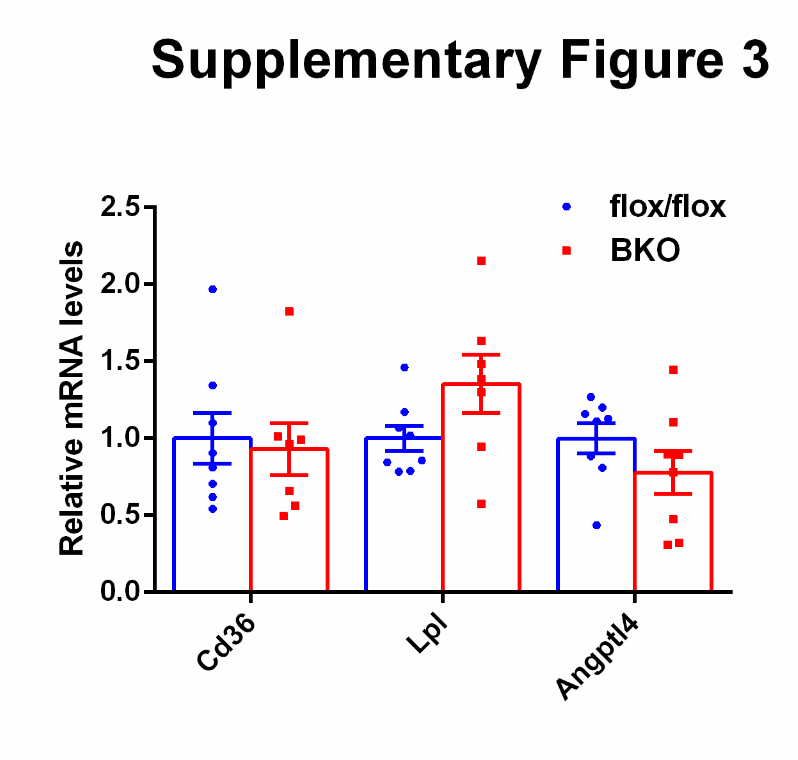


**Supplementary Figure 3. BAT-specific deletion of *Mettl3* does not affect the expression of lipid uptake associated genes in iBAT.**

Relative *Cd36*, *Lpl* and *Angptl4* mRNA levels were measure by qPCR assays in iBAT of 8-week-old *Mettl3*$^{flox/flox}$ and BKO mice (Cd36, Lpl in BKO, n = 7; Others, n=8 for each group).

Data represent the mean ± SEM. Source data are provided as a Source Data file.

# Supplementary Figure 4

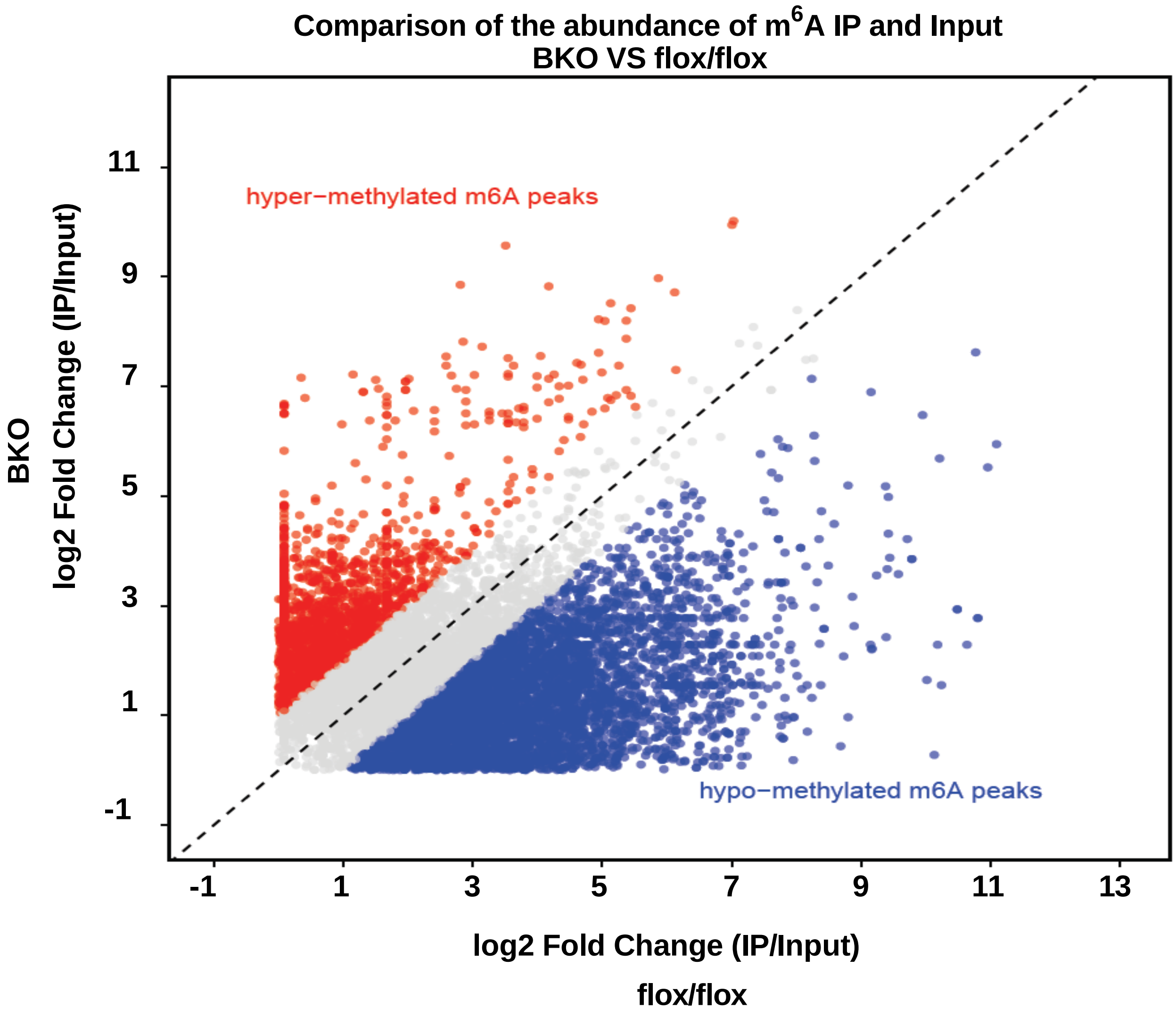


**Supplementary Figure 4. METTL3 is essential for mRNA m$^6$A modification in iBAT.**

The m$^6$ARIP-seq analysis of iBATs was performed in 8-week-old *Mettl3*$^{flox/flox}$ and BKO mice. Comparison of the abundance of m$^6$A IP and Input mRNAs (BKO VS flox/flox).

Supplementary Figure 5

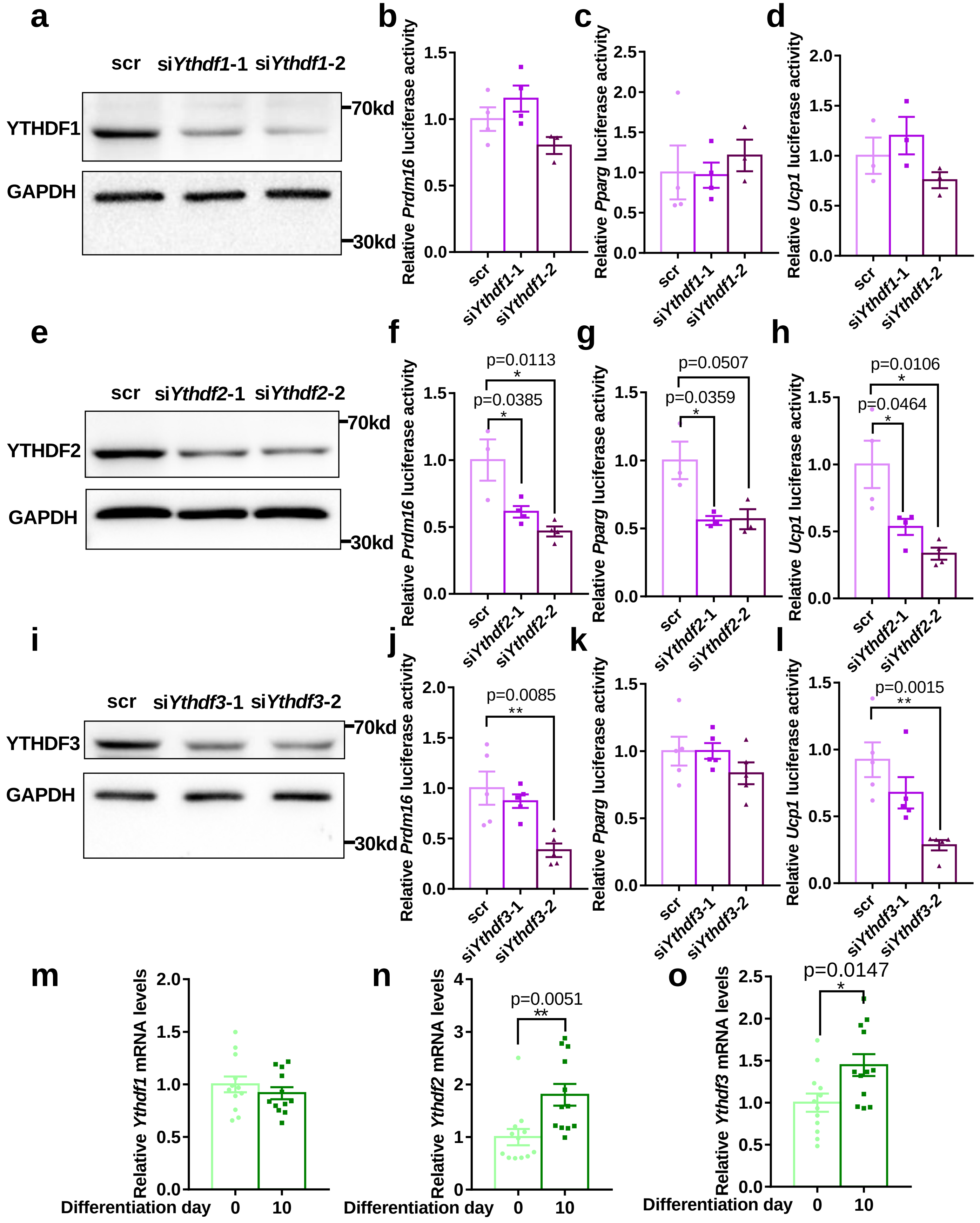

**Supplementary Figure 5. m$^6$A reader proteins regulate *Prdm16*, *Pparg* and *Ucp1* luciferase activity.**

Primary brown preadipocytes seeded in 24-well plates were co-transfected with pMIR-REPORT Luciferase vectors (*Prdm16*, *Pparg*, *Ucp1*), siRNAs (Scramble siRNA, si*Ythdf1*-1, si*Ythdf1*-2; Scramble siRNA, si*Ythdf2*-1, si*Ythdf2*-2; or Scramble siRNA, si*Ythdf3*-1, si*Ythdf3*-2) and β-galactosidase (β-Gal) reporter plasmid by X-tremeGENE siRNA Transfection Reagent for 24 hours. Cells were then induced to differentiate for 48 hours. (a, e, i) YTHDF1-3 and GAPDH protein levels were measured by immunoblotting. (b-d, f-h, j-l) Relative *Prdm16*, *Pparg* and *Ucp1* luciferase activity were measured and normalized to β-Gal levels (n=3-5 for each group). (m-o) The relative *Ythdf1-3* mRNA levels in mature primary brown adipocytes (10 days after differentiation) and preadipocytes (before differentiation) (n=12 for each group).

These cell culture experiments were repeated three times independently with similar results.

Data represent the mean ± SEM. Significance was determined by unpaired two-tailed Student's *t* test analysis.

*, $p < 0.05$. **, $p < 0.01$. Source data are provided as a Source Data file.

# Supplementary Figure 6

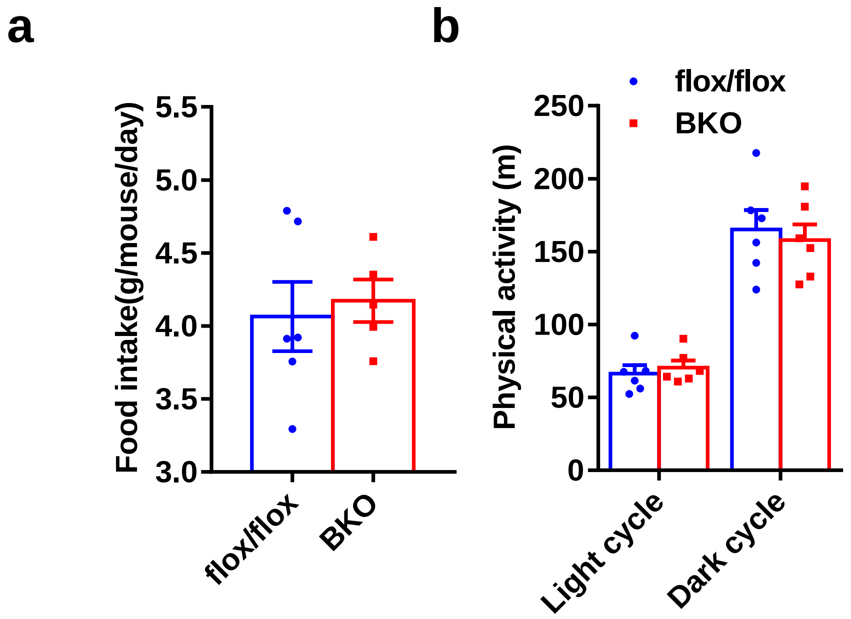


**Supplementary Figure 6.** ***Mettl3*** **deficiency in BAT does not affect food intake or physical activity.**

(a) The food intake of 8-week-old *Mettl3*$^{flox/flox}$ and BKO mice (*Mettl3*$^{flox/flox}$, n = 6; BKO, n = 5).

(b) The physical activity of 8-week-old *Mettl3*$^{flox/flox}$ and BKO mice (n = 6 for each group).

Data represent the mean ± SEM. Source data are provided as a Source Data file.

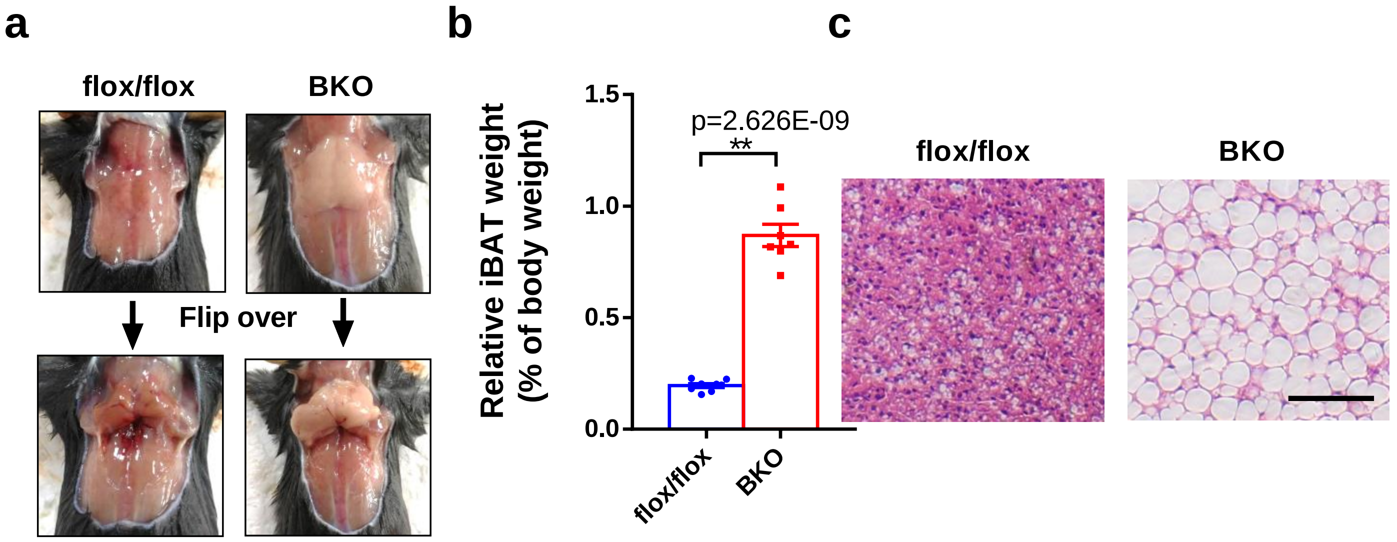


**Supplementary Figure 7. The morphology of iBAT in BKO mice under the cold exposure condition still appears abnormal, enlarged and "whitening".**

(a, b, c) Gross appearance, relative weight, and H&E staining of iBATs in *Mettl3*$^{flox/flox}$ and BKO mice ( 8 weeks) after acute cold exposure (4℃) for 6 h (b: *Mettl3*$^{flox/flox}$, n = 8; BKO, n = 7). The scale bar represents 100 µm. Data represent the mean ± SEM. Significance was determined by unpaired two-tailed Student's *t* test analysis. **, p< 0.01. Source data are provided as a Source Data file.

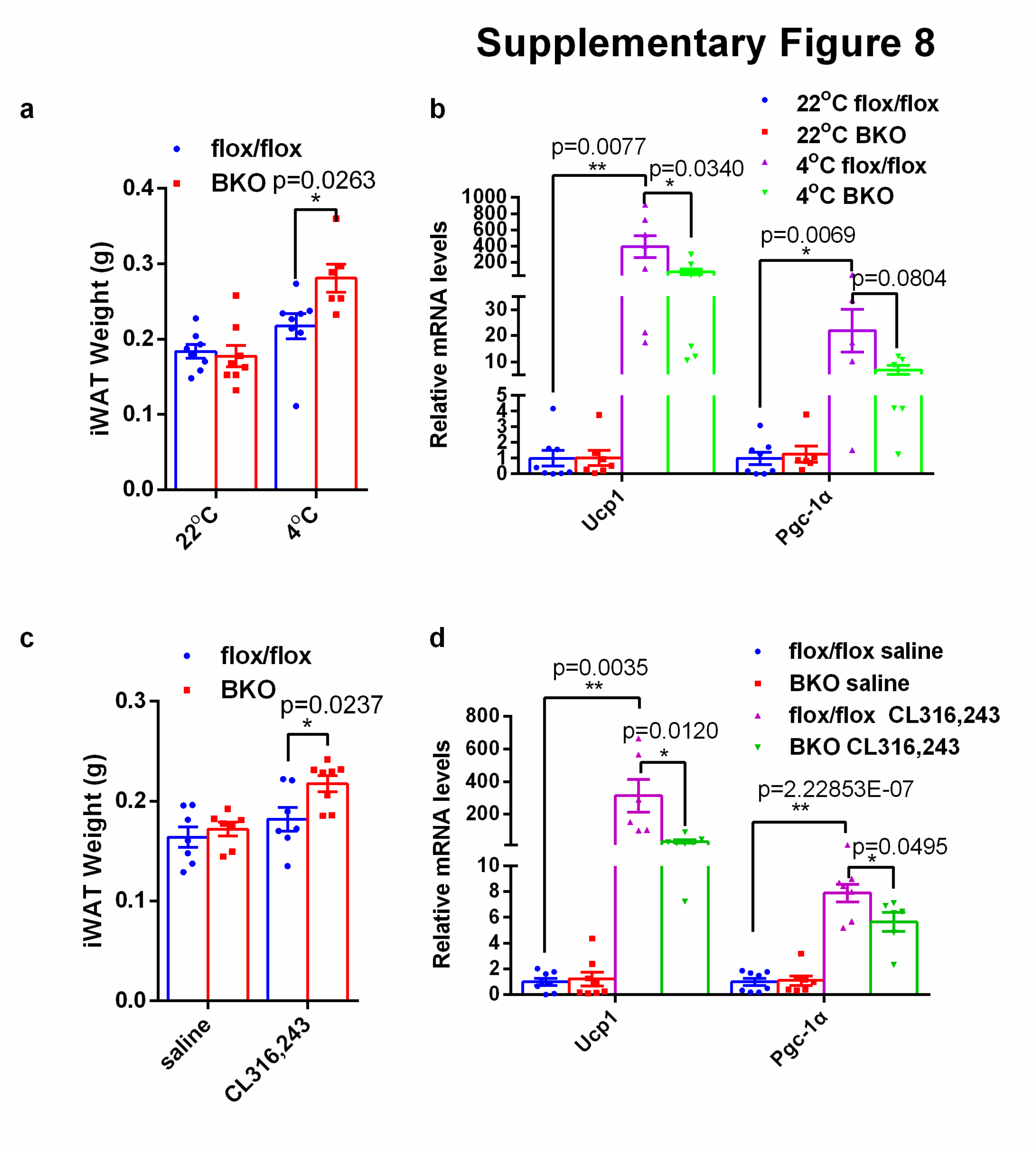


**Supplementary Figure 8. BAT-specific deletion of *Mettl3* impairs the browning of WAT in response to chronic cold exposure or the β-adrenergic agonist.**

BKO mice and flox/flox controls were exposed for chronic cold challenge (4℃ 7d) or injected intraperitoneally with CL316,243 for 4 days to induce browning of WAT. (a, c) The weight of iWAT was measured in BKO and flox/flox mice (n=6-8 for each group). (b, d) The relative *Ucp1* and *Pgc-1α* mRNA levels were measured by qPCR analysis in iWAT of BKO and flox/flox mice (n=5-8 for each group).

Data represent the mean ± SEM. Significance was determined by unpaired two-tailed Student's *t* test analysis.

*, $p < 0.05$.**, $p < 0.01$. Source data are provided as a Source Data file.

**Supplementary Table 1 Primers for qPCR**

| Genes | Forward | Reverse |
|---|---|---|
| Pparg2 | 5'-TGGGTGAAACTCTGGGAGATTC-3' | 5'-GAGAGGTCCACAGAGCTGATTCC-3' |
| Pgc-1α | 5'-TGGACGGAAGCAATTTTTCA-3' | 5'-TTACCTGCGCAAGCTTCTCT-3' |
| Adiponectin | 5'-GCACTGGCAAGTTCTACTGCAA-3' | 5'-GTAGGTGAAGAGAACGGCCTTGT-3' |
| Prdm16 | 5'-CAGCACGGTGAAGCCATTC-3' | 5'-GCGTGCATCCGCTTGTG-3' |
| Cebpb | 5'-TTATAAACCTCCCGCTCGGC-3' | 5'-TTCCATGGGTCTAAAGGCGG-3' |
| Ucp1 | 5'-TGGAAAGGGACGACCCCTAA-3' | 5'-CAGGAGTGTGGTGCAAAACC-3' |
| Adrb3 | 5'-CTATGCCAACTCCGCCTTCA-3' | 5'-GCCATCAAACCTGTTGAGCG-3' |
| Ndufa8 | 5'-GAGTTTATGCTGTGCCGCTG -3' | 5'-TACTCTGTGAAAGGCTCCGC-3' |
| Uqcrq | 5'-TTCAGCAAAGGCATCCCCAA-3' | 5'-CGACTGCTCAAACTCCTGGT-3' |
| Sdhb | 5'-GACGTCAGGAGCCAAAATGG-3' | 5'-CTCGACAGGCCTGAAACTGC-3' |
| Glut4 | 5'-TCTCCAACTGGACCTGTAAC-3' | 5'-TCTGTACTGGGTTTCACCTC-3' |
| Cox5b | 5'-AGAAGGGACTGGACCCATACA -3' | 5'-CCTTTGTGCAGCCAAAACCA-3' |
| Cebpa | 5'-CAAGAACAGCAACGAGTACCG-3' | 5'-GTCACTGGTCAACTCCAGCAC-3' |
| Cox6b1 | 5'-AACTACCTGGACTTCCACCG-3' | 5'-GGTACCACTCACACACGGAG-3' |
| Acta1 | 5'-CGACGGGCAGGTCATCA-3' | 5'-ACCGATAAAGGAAGGCTGGAA-3' |
| Myoglobin | 5'-CATGGACAGGAAGTCCTCATCG-3' | 5'-CTGTGAGCACGGTGCAACCATG-3' |
| MHC-1β | 5'-GAGGAAGAGTGAGCGGCG-3' | 5'-GCCGCAGTAGGTTCTTCCTGT-3' |
| MHC-IIa | 5'-TACAACCTCAAAGAGCGTTATGCA-3' | 5'-AAGGGTTGACGGTGACACAGA-3' |
| MEF2c | 5'-GAGCGTGCTGTGCGACTGT-3' | 5'-CGTGCGGCTCGTTGTACTC-3' |
| Casq2 | 5'-ACATCAAAGACCCACCCTACGT-3' | 5'-CGATGTGGATCCCATTCAAGT-3' |
| Tnnc1 | 5'-CCTGTGGTGCCTCCTTTGATT-3' | 5'-TGCGGTCTTTTAGTGCAATGAG-3' |
| ATGL | 5'-GAGGAATGGCCTACTGAACCAA-3' | 5'-AGGCTGCAATTGATCCTCCTC-3' |
| Fasn | 5'-TTGACGGCTCACACACCTAC-3' | 5'-CGATCTTCCAGGCTCTTCAG-3' |
| Srebp1 | 5'-AACGTCACTTCCAGCTAGAC-3' | 5'-CCACTAAGGTGCCTACAGAGC-3' |
| Cidea | 5'-ATCACAACTGGCCTGGTTACG-3' | 5'-TACTACCCGGTGTCCATTTCT-3' |
| Cpt1b | 5'-CATGTATCGCCGCAAACTGG-3' | 5'-GGTGCTGTAGCAAGTCTGTCT-3' |
| Cox6a | 5'-GAGGGTTCAGCTCGGATGTG-3' | 5'-GGTCTCTCGTGCTCTTCGTG-3' |
| Cox6b1 | 5'-AACTACCTGGACTTCCACCG-3' | 5'-GGTACCACTCACACACGGAG-3' |
| Cox7a1 | 5'-TCTTCCAGGCCGACAATGAC-3' | 5'-GCCCAGCCCAAGCAGTATAA-3' |
| nLPL | 5'-GGATGGACGGTAAGAGTGATTC-3' | 5'-ATCCAAGGGTAGCAGACAGGT-3' |
| mtND1 | 5'-GTGGCTCATCTACTCCACTGA-3' | 5'-TCGAGCGATCCATAACAATAA-3' |
| Pfkm | 5'-GGCGGAGGAGAGCTAAAACT -3' | 5'-CCCTGACGGCAGCATTCATA-3' |
| Cpt2 | 5'-CAAAAGACTCATCCGCTTTGTTC-3' | 5'-CATCACGACTGGGTTTGGGTA-3' |
| MG2 | 5'-GCTGTGGCGGTAGTGGAA-3' | 5'-ATGAGGGCCTTGGGTGTG -3' |
| HSL | 5'-GTGGTGTGTAACTAGGATTGACTCT-3' | 5'-GAACGCTGAGGCTTTGATCTTG-3' |
| aP2 | 5'-AAGGAAAGTGGCAGGCATGG-3' | 5'-CACGCCCAGTTTGAAGGAAATC-3' |

| | | |
|---|---|---|
| 36B4 | 5'-AAGCGCGTCCTGGCATTGTCT-3' | 5'-CCGCAGGGGCAGCAGTGGT-3' |
| Prdm16-m6A | 5'-AAAAGGACCCAGGTAGCCCT-3' | 5'-GGACCCGTGTCCCAACTATC-3' |
| PPARg-m6A | 5'-GACAGACCTCAGGCAGATCG -3' | 5'-AAGGAACACGTTGTCAGCGG-3' |
| UCP1-m6A | 5'-CAGGACGGTGCCCTGTATTT-3' | 5'-TCGTGGTCTCCCAGCATAGA-3' |
| Lpl | 5'-AGAAGGGAAAGGACTCAGCAG-3' | 5'-TCAAACACCCAAACAAGGGTA-3' |
| Angptl4 | 5'-ACTTCAGATGGAGGCTGGAC-3' | 5'-TCCGAAGCCATCCTTGTAGG-3' |
| Cd36 | 5'-GGAGTGGTGATGTTTGTTGCT-3' | 5'-GCACACACCACCATTTCTTCT-3' |
| Ythdf1 | 5'-CTGCAGTTAAGACGGTGGGT-3' | 5'-TAGCAATGGCTGCCCATGAA-3' |
| Ythdf2 | 5'-AGCCAATGAGGAAAGGGCATT-3' | 5'-CTCCCCAAACACAGAGACTCAA-3' |
| Ythdf3 | 5'-TGTTCTATCTTGATTTGACTTTGCT-3' | 5'-ATAGCTGTTATTCTGATTTGTCTGG-3' |
